# Topological memory of nonreciprocal scattering singularities

**DOI:** 10.1038/s41467-026-77640-8

**Published:** 2026-09-25

**Authors:** Haoye Qin, Zhe Zhang, Wenjing Lv, Jue Li, Junda Wang, Zijin Yang, Qinghua Song, Romain Fleury

**Affiliations:** 1 Tsinghua Shenzhen International Graduate School, Tsinghua University, Shenzhen, China; 2 Department of Electrical Engineering, Ginzton Laboratory, Stanford University, Stanford, CA USA; 3 Laboratory of Wave Engineering, School of Electrical Engineering, EPFL, Lausanne, Switzerland; 4 School of Physics and Optoelectronic Engineering, Beijing University of Technology, Beijing, China

**Keywords:** Electronics, photonics and device physics, Photonic devices

## Abstract

Non-Hermitian systems host a wealth of exotic phenomena, yet their temporal topology—particularly in time-reversal-symmetry-broken conditions—remains largely unexplored. Here, we experimentally demonstrate topological memory emerging from hysteresis-driven dynamics in a nonreciprocal microwave scattering system. The scattering singularity, encompassing both backscattering zeros and nonreciprocal exceptional points (EPs), serves as the cornerstone of this effect. Magnetic hysteresis induces a history-dependent trajectory of the complex scattering parameter that winds around such singularities, giving rise to quantized topological charges and sharp transitions when crossing them. At the limit of infinite nonreciprocity, backscattering zeros facilitate access to EPs, drastically easing their realization and yielding enhanced frequency combs near the transition. Extending these dynamics to free-space broadcasting and circulator-based topological insulators, we demonstrate nonreciprocity-assisted transport and long-distance preservation of scattering singularities. Our results uncover the interplay among singularity, nonreciprocity, and Floquet engineering, establishing a unified platform for exploring temporal topology and in-memory non-Hermitian photonics.

## Abstract (web summary)

Wave systems with memory can exhibit topological effects that depend on their history. Here, the authors demonstrate hysteresis-driven topological memory in a nonreciprocal microwave system, linking scattering zeros to exceptional points and preserving them over long distances.

## Introduction

Over recent decades, non-Hermitian physics has witnessed a surge of interest^1,2^, unveiling a variety of scattering singularities that govern the behavior of waves in open systems. Among them, reflection zeros, where outgoing fields vanish, and exceptional points (EPs), where eigenvalues and eigenstates coalesce, represent two fundamental classes of singularities^3–7^. These singularities underlie a range of striking phenomena, including nontrivial topology^6,8^, unidirectional invisibility^9,10^, enhanced sensitivity^11–13^, and half-charged bulk Fermi arcs^14^. Initially conceived as a theoretical framework, non-Hermitian physics has matured into a broad experimental field spanning optics^15,16^, acoustics^17^, magnons^18^, and ultracold atoms^19,20^. EPs have enabled new mechanisms for wave manipulation in coupled-mode systems^21,22^, photonic crystals^23^, polarization converters^24^, and scattering matrices^25–27^. Their non-adiabatic encirclement has also revealed geometric mode transfer and chirality in synthetic and dynamically tuned platforms^28–30^, highlighting the central role of scattering singularities in modern wave physics. With the rising explorations into time-variant systems and temporal interfaces^31–35^, new degrees of freedom emerge through time-delayed Floquet dynamics in non-Hermitian and nonreciprocal platforms, opening opportunities to explore temporal topology and its transitions at time-domain interfaces. In particular, nonreciprocity—typically induced by magnetic bias—naturally introduces hysteresis and a temporal lag, while material absorption provides the intrinsic loss necessary for non-Hermitian operation. These ingredients can be seamlessly integrated into a modulated microwave circulator, offering an ideal arena to probe the interplay between memory and nonreciprocity. Yet, despite its conceptual richness, the temporal topological dynamics of memory-based dynamic systems has remained largely untapped.

Here, we experimentally demonstrate a topological memory effect arising from hysteresis-driven dynamics in a nonreciprocal microwave scattering system. The system exhibits scattering singularities—encompassing both backscattering zeros and nonreciprocal exceptional points—that govern the quantized winding of a complex scattering parameter. Magnetic hysteresis generates history-dependent loops in the complex plane, which can encircle singularities and produce quantized topological charges and fast transitions when the loop crosses a singular point. We further reveal that, in the limit of infinite nonreciprocity, backscattering zeros can evolve into exceptional points, thereby easing their realization and yielding enhanced frequency combs near the transition. Extending these dynamics to free-space broadcasting and circulator-based nonreciprocal topological insulators, we demonstrate nonreciprocity-assisted transport and preservation of scattering singularities over long distances. Our findings uncover a unified picture of nonreciprocal scattering singularities, bridging zeros and exceptional points through hysteresis-induced temporal topology. This establishes a general framework for in-memory non-Hermitian systems, where nonreciprocity, topology, and Floquet engineering converge to produce novel dynamic phenomena.

## Results

### Concept of topological memory

**Fig. 1:**
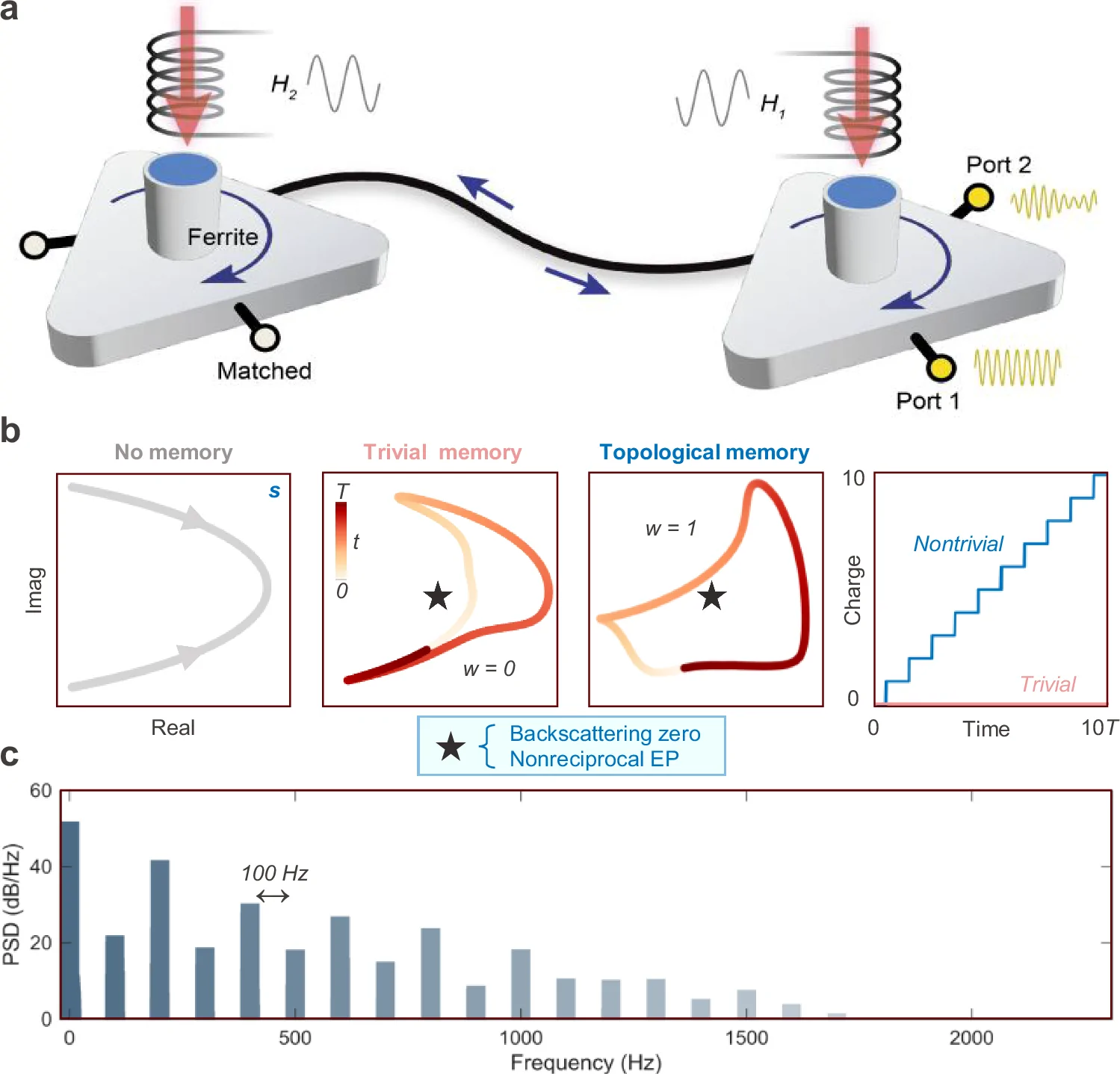
Topological memory of nonreciprocal scattering singularities. **a** Schematic setup of hysteretic memory in a nonreciprocal and non-Hermitian scattering system composed of two cascaded circulators connected by a transmission line. Each ferrite circulator is periodically modulated by external magnetic fields *H*_1,2_ proportional to applied voltages (sinusoidal with frequencies *f*_1,2_). The scattering parameter at a fixed microwave frequency is monitored at two ports, with all remaining ports matched. Each circulator consists of a ferrite post sandwiched between two permanent magnets that generate a constant bias field. The external modulation introduces magnetic hysteresis, producing memory loops of the complex scattering parameter. **b** Illustration of the no-memory path and the trivial and topological windings induced by hysteretic evolution of the complex scattering parameter *s*. The singularity marked by the star represents a backscattering zero or a nonreciprocal exceptional point (EP). Over one modulation period *T*, topological and trivial memory loops acquire winding charges *w* of 1 and 0, respectively, with the total charge accumulating over longer durations (right panel). **c** Measured power spectral density (PSD) spectrum of *S*_21_ showing hysteresis-induced frequency combs under *f*_1,2_ = 100 Hz modulation with a 5-GHz microwave excitation.

We demonstrate the topological memory within a non-Hermitian scattering system composed of two cascaded, nonreciprocal circulators subjected to external magnetic fields (Fig. 1a). The system is connected through transmission lines and described by an effective two-port scattering matrix, with all auxiliary circulator ports terminated by 50-*Ω* matched loads as in standard multiport scattering measurements. These matched ports suppress uncontrolled reflections, making the reduced two-port matrix non-unitary together with intrinsic loss. Each circulator incorporates lossy ferrite posts under a static bias field provided by inner permanent magnets (inducing Zeeman splitting that lifts the degeneracy between two counter-propagating modes). The application of time-periodic magnetic fields gives rise to magnetic hysteresis, resulting in distinct memory loops of the scattering parameter in the complex plane, and a history dependency that can be leveraged to create a memory^36–38^. It is important to note that the loop is enabled only by the magnetic hysteresis, and would degenerate to a line if such past-state memory were absent (Fig. 1b). These memory loops exhibit topological or trivial characteristics depending on their encirclement or exclusion of the singular point (star in Fig. 1b), quantified by a winding number $w=\frac{1}{2\pi }\int_{0}^{T}\frac{d}{d\tau }\arg [s(\tau )]\,d\tau,$ where *s*(*τ*) is the selected complex scattering parameter evolving over one modulation period *T*. The singularity, regarded as a topological defect, can manifest either as a backscattering zero with *s* = *S*_21_ = 0 ^39^, or as a nonreciprocal EP exhibiting non-Hermitian geometry with braiding topology^6^. Over extended periods encompassing multiple cycles, the topologically nontrivial loop of *w* = 1 accumulates a large winding charge, whereas the trivial loop remains at zero. Practically, the loop must have sufficient area and lag to avoid collapsing into a retraced line, while loss is needed to make the scattering matrix non-unitary and allow zeros or EPs. Nonreciprocity arises from time-reversal-symmetry breaking in magnetically biased ferrites and makes scattering direction dependent. It enables a directional zero with finite reverse transmission, creating an infinite-nonreciprocity limit that relaxes the constraints for reaching an EP and supports memory-driven braiding transitions. The hysteretic memory of the transmitted signal can be experimentally studied by looking at its power spectral density, which is characterized by a frequency comb of harmonics separated by the modulation frequency (Fig. 1c).

### Topological memory of scattering zeros

**Fig. 2:**
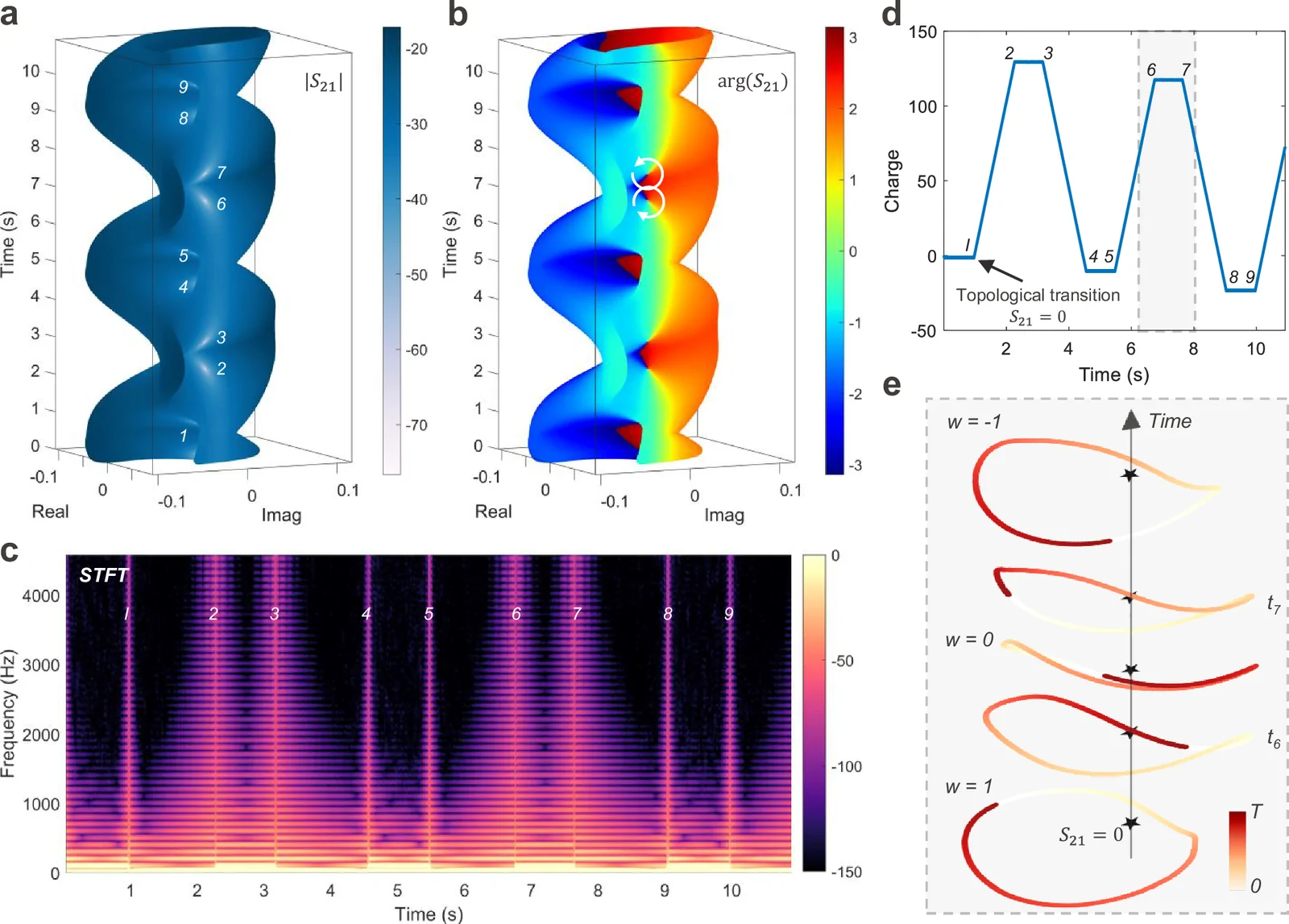
Topological memory of backscattering zero. **a**, **b** Amplitude (**a**) and phase (**b**) maps of scattering parameter *S*_21_ over 11 s with asynchronous modulation *f*_1,2_ = 100, 100.4 Hz. The numbers 1-9 mark topological transition points where the hysteretic memory loop crosses the backscattering zero, switching between trivial and topological winding regimes. **c** The corresponding short-time Fourier transformation spectrum shows enhanced combs in the vicinity of the singularity. **d** Evolution of accumulated topological charge over time, which undergoes topological transition when the loop touches *S*_21_ = 0. **e** Memory loops within one period starting at five time instants with trivial winding, topological winding, and topological transition near singularities 6 and 7.

With the hysteretic memory mechanism and resultant loss-and-nonreciprocity modulation at hand, we start with demonstrating topological transitions of the complex transmission *S*_21_, that is, the crossing of a backscattering zero. We employ an asynchronous modulation for two fields at frequencies of *f*_1,2_ = 100, 100.4 Hz, which is chosen as a practical compromise: slow enough to ensure stable hysteresis, yet fast enough to capture multiple memory cycles within the measurement window. This creates a beating mechanism that enables an adiabatic periodic deformation of the winding loop, potentially driving temporal topological transitions by closely crossing the singularity *S*_21_ = 0. Figure 2a, b show the dynamic evolution of the amplitude and phase of *S*_21_ within the complex plane over time. Clear manifestations of zero singularities (labeled from 1 to 9) and associated temporal phase vortices are experimentally observed. We note that the temporal profile of the complex *S*_21_ is induced by hysteretic memory, and the topological charge can be viewed from the perspective of monotonically increasing time (memory loop) (Fig. 2b). It is expected that the singularity crossing is extremely brief in time, which is confirmed by looking at the short-time Fourier transform (STFT) of the transmitted signal in Fig. 2c. On the occurrence of *S*_21_ = 0, we observe an enhanced broadband frequency comb spanning over 4 kHz, at the locations of labels 1-9 in the figure (see also Supplementary Fig. 1 and Fig. 2). This verifies the fast transition and strong harmonic generation enabled by the temporal memory and topological singularity. To demonstrate more clearly this memory loop and its topological transition in the vicinity of the singularity, the evolution of the topological charge is shown in Fig. 2d. Figure 2e plots the memory loop over one period near the singularities 6 and 7 for five typical cases: topological memory (clockwise and counterclockwise encircling *w* = ± 1), trivial memory (*w* = 0), and topological transition (crossing zero).

### Topological memory of EPs

**Fig. 3:**
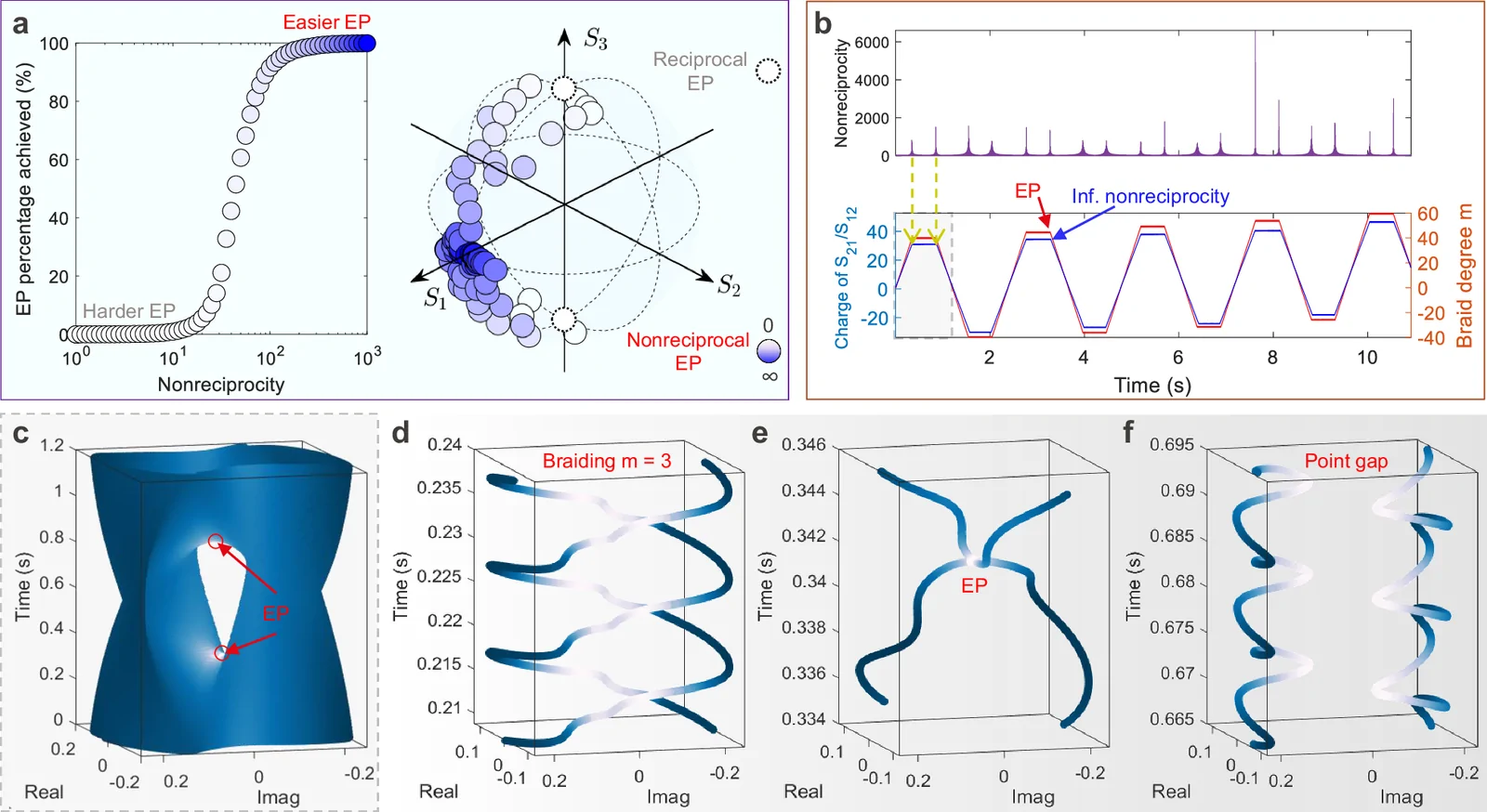
Topological memory of nonreciprocal EPs. **a** Theory on nonreciprocal EP. Large nonreciprocity is expected to make the system approach EP more easily. The corresponding coalesced eigenstates at EP are plotted on Poincaré sphere where reciprocal EP must lie at either of the two poles ([1±*j*]^*T*^) and nonreciprocal EPs span over the whole sphere. For nearly infinite nonreciprocity, EP eigenstates occur near [10]^*T*^ or [01]^*T*^ on the equator. The statistical results are extracted from 10^5^ random scattering matrices for each value of nonreciprocity. The dot’s color represents the level of nonreciprocity. **b** Experimental observation of topological memory of nonreciprocal EPs near infinite nonreciprocity (*S*_21_ → 0). Infinite nonreciprocity and EP occur at topological transitions of *S*_21_/*S*_12_ winding charge and braid degree, respectively. **c** Temporal geometry of scattering eigenvalues for the shaded region in **b**. EP occurs at the two saddle centers, representing the transition between braiding and non-Hermitian point gap. **d**–**f** Detailed view of three typical cases of braiding **d**, EP **e**, and point gap **f** over time using hysteretic memory.

The backscattering zero has another implication in a nonreciprocal scattering system: it corresponds to the limit of infinite nonreciprocity (lossy perfect circulation), where one transmission coefficient vanishes while the opposite channel remains finite, e.g., *S*_21_ → 0 and *S*_12_ ≠ 0. Physically, the biased ferrite circulator lifts the degeneracy of counter-propagating modes and creates directional circulation; together with loss and impedance mismatch, this allows destructive interference in one channel while maintaining finite reverse transmission. In the following, we show that infinite nonreciprocity is markedly advantageous in realizing an exceptional point. In Fig. 3a, we run a statistical study involving generating random complex scattering matrices with different values of nonreciprocity and determining the number of EPs achieved in these random matrices. The EP condition for a given matrix is obtained analytically in Supplementary Note 2 and used in our analysis. We find that higher nonreciprocity contributes to matrix degeneracy and an easier realization of EP, in contrast with reciprocal systems (see Supplementary Note 2). Correspondingly, the degenerate eigenstates at the EP deviate from the fixed circular states ([1 ±*j*]^*T*^) characteristic of reciprocal EPs^10^, extending instead over the entire Poincaré sphere. With nearly infinite nonreciprocity, the EP eigenstate will lie close to [10]^*T*^ or [01]^*T*^ on the equator (see detailed derivation in Supplementary Note 2). This prediction indicates that scattering EP should be more readily accessed near the infinite nonreciprocity condition of *S*_21_ = 0. In experiment, we confirm this point as shown in Fig. 3b with the evolution of nonreciprocity, memory charge of *S*_21_/*S*_12_, and braid degree over time. The braid degree characterizing how many times two eigenvalues braid^6,40^ can be defined as $m=\int_{0}^{t}\frac{d\tau }{2\pi i}\frac{d}{d\tau }\,{\mbox{ln}}\,\,\,{\mbox{det}}\,(S(\tau )-{E}_{r}I),$ where *S* is the scattering matrix, *E*_*r*_ is a chosen reference, and *I* is the identity matrix. We note that topological transitions of charge (infinite nonreciprocity) and braid degree (exact EP) occur at the same instant as the maximum nonreciprocity pulses. Figure 3c plots the trajectory of eigenvalues in the complex plane over a time of 1.2 s, forming a temporal geometry. This trajectory reveals two nonreciprocal EPs, positioned at two saddle centers, which correspond to the transition between non-Hermitian braiding and point gap (no braiding). Zoomed-in temporal view shows the three typical cases: non-Hermitian braiding (Fig. 3d), nonreciprocal EP (Fig. 3e), and point gap (Fig. 3f) induced by hysteretic memory process (the corresponding evolution of eigenstates on Poincaré sphere is indicated in Supplementary Fig. 5). Largely enhanced memory combs and transient phenomena in the vicinity of nonreciprocal EPs are observed in STFT spectrum of Supplementary Fig. 3. The overall braiding process is shown in the Supplementary Movie. We are also able to achieve extremely large braid degrees within a short time by using synchronized modulation that only encircles EP without transition (see Supplementary Fig. 7).

### Remote reconstruction of EPs

**Fig. 4:**
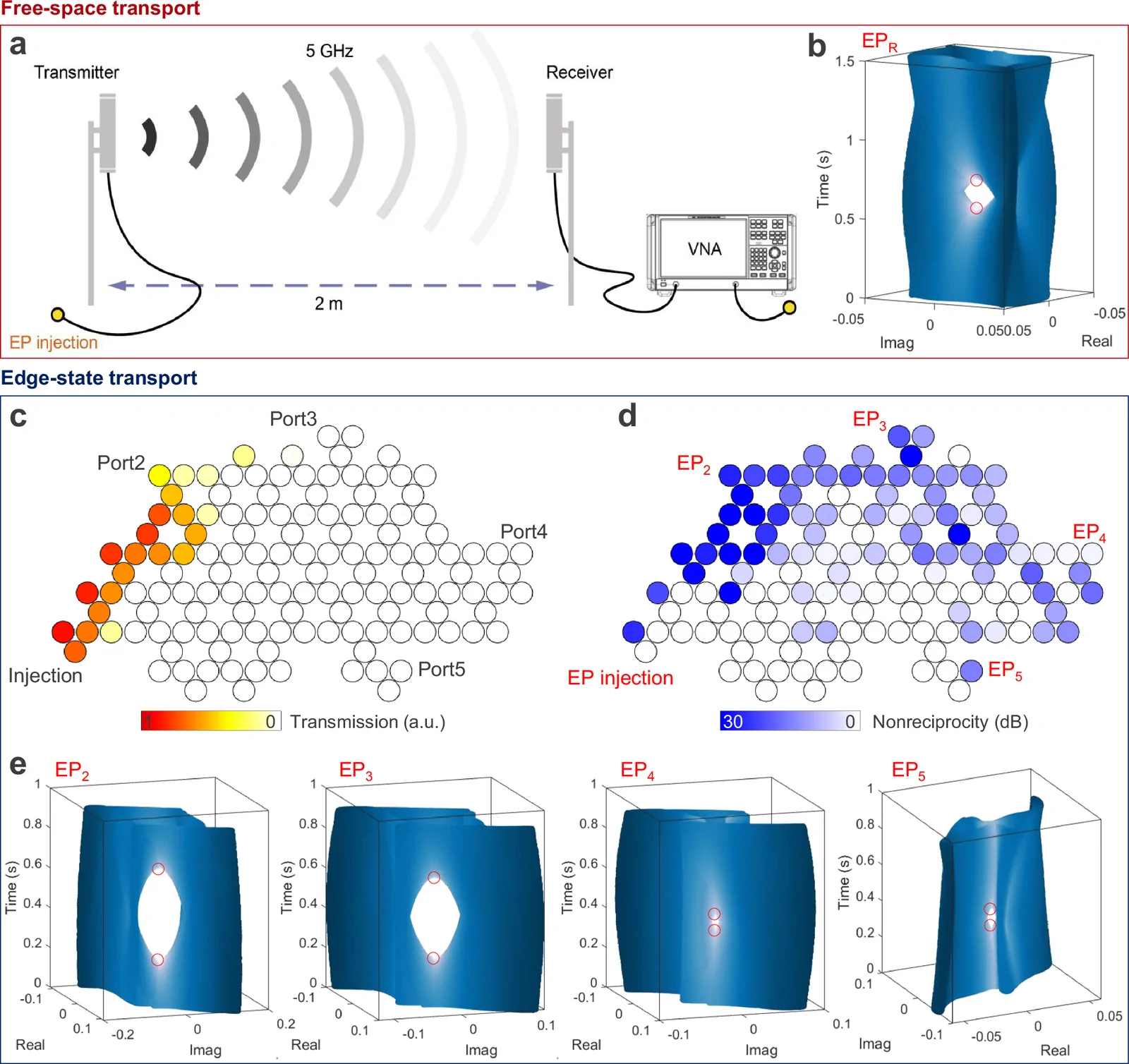
Experimental remote-reconstruction of nonreciprocal in-memory EPs. **a**, **b** Free-space EP transport. **a** Experimental setup for free-space transport of EP with transmitting and receiving antennas. **b** Measured temporal geometry of scattering eigenvalues indicates two EPs (red circles). **c**–**e** Nonreciprocity-preserved topological edge-state EP transport. Experimentally realized disordered topological network with circulators as scattering nodes and connected by transmission lines. When injected at one port, the wave propagates along the boundary of the nonreciprocal topological insulator but largely decays due to intrinsic loss. **c** The transmission unavoidably becomes undetectable for ports 3-5. **d** Considerably large nonreciprocity can be preserved at distant ports. Hysteretic memory of nonreciprocal EPs is achieved at ports 2–5. **e** Temporal trajectories of scattering eigenvalues reveal two EPs (red circles) at each port.

Finally, we demonstrate remote reconstruction and transport of nonreciprocal in-memory EP signatures. An EP is a property of an effective scattering matrix rather than of the propagating signal itself. Here, the time-dependent complex scattering response encoding the EP topology is transmitted through free space or a guided-wave network and reconstructed remotely. This shows that non-Hermitian topological memory can persist beyond a local device, suggesting potential applications in remote readout, distributed sensing, and information encoding based on temporal scattering trajectories. For the free-space transport, this is achieved using two antennas—one for transmitting and one for receiving the time-domain nonreciprocal memory signals—placed two meters apart (Fig. 4a). The topological memory of EP is first generated using the temporal modulation setup in Fig. 1a, and its output is coupled to a transmitting antenna for free-space broadcasting at 5 GHz. The receiving antenna successfully retrieves the temporal response, as shown in Fig. 4b, verifying nonreciprocity-assisted EP transport in free space. This observation demonstrates that topological memory, once imprinted around an EP, can be remotely reconstructed without direct electrical connection, opening a route to long-range transfer of non-Hermitian states.

We further extend the concept of remote reconstruction onto guided-wave systems by integrating the topological memory of EPs with nonreciprocity-preserved topological edge states that originate from anomalous Floquet topology^41–43^. These chiral edge channels enable spatially robust wave transport immune to disorder and geometric imperfections (See Supplementary Note 4 for the underlying anomalous Floquet topology, including band structure, invariant, edge states, and simulated fields). Experimentally, we implement the topological lattice using a circulator network interconnected by microstrip transmission lines on a printed circuit board (see Supplementary Fig. 10), where deliberate boundary irregularities are introduced to challenge the robustness of the edge states. In Fig. 4c, measured transmission maps under static conditions reveal energy propagation confined to the network’s boundary, confirming the spatial existence of a topological edge state. However, radiative and absorption losses lead to amplitude decay and the signal is not resolved on the map beyond port 2. In contrast, the nonreciprocal character of the network is preserved even after very long propagation distances and is detectable at distant ports as shown in Fig. 4d. Beyond a mere static, unidirectional edge flow, the strong nonreciprocity enables the hysteretic EPs to persist after long-range propagation of temporal memory signals along the network boundary. By connecting port 2 of the temporal modulation setup (Fig. 1a) to the input of the topological insulator network and probing the transmitted waves at ports 2–5 respectively, we experimentally demonstrate the emergence of nonreciprocity-preserved topological edge-state EPs over time with intrinsic memory at each port (Fig. 4e). Although distortions appear in the temporal trajectories, the exceptional points at the saddle centers remain clearly observable. The experiment tests whether the complex temporal scattering trajectory that defines the EP topology can survive propagation through an independent, lossy, and disordered system. Although ordinary transmission decays strongly at remote ports, the nonreciprocal edge channel preserves the directional amplitude and phase response sufficiently to recover the EP-type eigenvalue coalescence and braiding, enabling long-distance readout of non-Hermitian memory topology. This observation also indicates a degree of tolerance to noise and parameter drift: continuous distortions of the received trajectory do not change the winding or braiding topology unless the loop crosses the singularity or the signal-to-noise ratio becomes too low to resolve the complex trajectory. Experimentally, the main sources of variation are ferrite-bias drift, modulation instability, propagation loss, and receiver noise, but the EP signatures were repeatably observed under the same modulation conditions. Therefore, we experimentally demonstrate long-range transport of non-Hermitian scattering singularities, enabled by topologically protected nonreciprocal channels in both spatial and temporal domains.

## Discussion

Although our experiment uses ferrite circulators, topological memory is not restricted to magnetic hysteresis. The key requirement is a history-dependent internal variable that drives a complex scattering parameter around a non-Hermitian singularity. Similar effects may therefore arise in memristive, thermo-optic, phase-change, optomechanical, ferroelectric, or nonlinear metamaterial systems. Ferrites are used here because hysteresis, loss, and nonreciprocity coexist naturally, while nonreciprocal EP memory in other platforms would require an additional time-reversal-symmetry-breaking mechanism, such as spatiotemporal modulation, active circuits, or synthetic gauge fields.

Another promising direction is to extend hysteresis-induced topological memory to nonreciprocal higher-order EPs. In such systems, a memory loop of the full scattering matrix could encircle or cross a higher-order degeneracy, producing higher-order eigenvalue permutations and more complex braid topology. Nonreciprocity may facilitate this extension by enlarging the accessible space of EP eigenstates, for example in multiport circulator networks, coupled resonator arrays, or active spatiotemporally modulated circuits.

In conclusion, we have demonstrated a hysteresis-induced topological memory effect in a nonreciprocal and non-Hermitian scattering system. The backscattering singularity serves as a topological defect around which the complex scattering parameter winds, generating quantized and cumulative topological charges. Importantly, this scattering singularity provides a natural pathway toward realizing nonreciprocal EPs, which act as topological transition points between non-Hermitian braiding and non-braiding regimes. We have also extended this concept of topological memory to both free-space and guided-wave transport of EPs within a circulator-based nonreciprocal topological insulator, demonstrating the long-distance preservation of scattering singularities assisted by nonreciprocity. These results establish a unified experimental framework that connects singularity, nonreciprocity, topology, and Floquet engineering, enabling the realization of in-memory transient EPs and temporal topological transitions.

Given the growing interest in Floquet systems and synthetic dimensions^44,45^, our work offers a new mechanism for inducing lag-driven temporal topology in photonic scattering and enriches the landscape of non-Hermitian degeneracies through nonreciprocity-enabled transient phenomena. Future directions include exploring temporal memory effects in other topological singularities or defects^39,46,47^, and extending topological boundary states into hysteresis-induced synthetic dimensions^48,49^, paving the way for controllable topological transitions and dynamic topology switching^42,50,51^.

## Methods

### Experimental setups

The circulator is a surface mount microwave circulator, designed from a Y-shaped strip line on a printed circuit board. The three ports are placed 120^∘^ apart from each other such that they are iso-spaced. The printed circuit board is sandwiched between two pieces of ferrite. Without magnetic fields, the Y-junction strip line supports two degenerate modes at *f*_0_: right and left-handed. To bias it, two internal magnets are fixed outside, providing the required magnetic field of 50 kA/m = 628 Oe, normal to the printed circuit board and polarizing the ferrite, therefore lifting the initial degeneracy, with chiral modes at *f*_+_ and *f*_−_. An electromagnet is placed above the circulator and a periodic modulation of voltage signal is applied to it with (${V}_{1,2}(t)={V}_{0}+{V}_{m}\sin (2\pi {f}_{1,2}t)$), generating a time-varying magnetic field normal to the circulator’s top surface. Two ports are connected to a vector network analyzer (VNA, Rohde & Schwarz), with all other ports terminated by matched loads. For time-domain measurements, the VNA provides a constant-amplitude continuous-wave microwave input at a fixed carrier frequency, typically near 5 GHz, while temporal variation is produced by low-frequency magnetic modulation over the circulator. In the free-space transport configuration, a pair of 5 GHz antennas are connected to the respective transmitter and receiver ports to establish the radiative link. For the field map measurements of Fig. 4c, d, we connect the signal input port of the measured network to VNA port 1, while perfectly matching the other ports of the network. We manually probe the field at the middle of the microstrip lines by using a coaxial probe, which is connected to VNA port 2.

## Supplementary information

Supplementary Movie

Supplementary Information

Description of Additional Supplementary Files

Transparent Peer Review file

## Data Availability

The data that support the findings of this study are presented within the main text and Supplementary Information.

## References

[1] Ashida, Y., Gong, Z. & Ueda, M. Non-Hermitian physics. *Adv. Phys.* **69**, 249–435 (2020).

[2] El-Ganainy, R., Khajavikhan, M., Christodoulides, D. N. & Ozdemir, S. K. The dawn of non-Hermitian optics. *Commun. Phys.* **2**, 1–5 (2019).

[3] Miri, M.-A. & Alù, A. Exceptional points in optics and photonics. *Science* **363**, eaar7709 (2019). 10.1126/science.aar7709 30606818

[4] Özdemir, S. K., Rotter, S., Nori, F. & Yang, L. Parity-time symmetry and exceptional points in photonics. *Nat. Mater.* **18**, 783–798 (2019). 10.1038/s41563-019-0304-9 30962555

[5] Li, A. et al. Exceptional points and non-Hermitian photonics at the nanoscale. *Nat. Nanotechnol.* **18**, 706–720 (2023). 10.1038/s41565-023-01408-0 37386141

[6] Ding, K., Fang, C. & Ma, G. Non-Hermitian topology and exceptional-point geometries. *Nat. Rev. Phys.* **4**, 745–760 (2022).

[7] Qin, H. et al. Photonic exceptional points in engineered materials and their emerging applications. *Nat. Rev. Mater.* **11**, 453–468 (2026).

[8] Said Ergoktas, M. et al. Localized thermal emission from topological interfaces. *Science* **384**, 1122–1126 (2024). 10.1126/science.ado0534 PMC7616096 38843319

[9] Feng, L. et al. Demonstration of a large-scale optical exceptional point structure. *Opt. Express* **22**, 1760–1767 (2014). 10.1364/OE.22.001760 24515183

[10] Huang, Y., Shen, Y., Min, C., Fan, S. & Veronis, G. Unidirectional reflectionless light propagation at exceptional points. *Nanophotonics* **6**, 977–996 (2017).

[11] Park, J.-H. et al. Symmetry-breaking-induced plasmonic exceptional points and nanoscale sensing. *Nat. Phys.* **16**, 462–468 (2020).

[12] Wiersig, J. Review of exceptional point-based sensors. *Photonics Res.* **8**, 1457–1467 (2020).

[13] Xu, J. et al. Single-cavity loss-enabled nanometrology. *Nat. Nanotechnol.* pages 1–6, July 2024. 10.1038/s41565-024-01729-8 39020101

[14] Zhou, H. et al. Observation of bulk Fermi arc and polarization half charge from paired exceptional points. *Science* **359**, 1009–1012 (2018). 10.1126/science.aap9859 29326118

[15] Parto, M., Liu, Y. G. N., Bahari, B., Khajavikhan, M. & Christodoulides, D. N. Non-Hermitian and topological photonics: optics at an exceptional point. *Nanophotonics* **10**, 403–423 (2021).

[16] Goldzak, T., Mailybaev, A. A. & Moiseyev, N. Light Stops at Exceptional Points. *Phys. Rev. Lett.* **120**, 013901 (2018). 10.1103/PhysRevLett.120.013901 29350937

[17] Wang, X., Fang, X., Mao, D., Jing, Y. & Li, Y. Extremely Asymmetrical Acoustic Metasurface Mirror at the Exceptional Point. *Phys. Rev. Lett.* **123**, 214302 (2019). 10.1103/PhysRevLett.123.214302 31809135

[18] Wang, C. et al. Enhancement of magnonic frequency combs by exceptional points. *Nat. Phys.* **20**, 1139–1144 (2024).

[19] Zhang, J. et al. Exceptional point and hysteresis trajectories in cold Rydberg atomic gases, August 2024. 10.1038/s41467-025-58850-y PMC11994797 40223112

[20] Xu, Y., Wang, S.-T. & Duan, L.-M. Weyl Exceptional Rings in a Three-Dimensional Dissipative Cold Atomic Gas. *Phys. Rev. Lett.* **118**, 045701 (2017). 10.1103/PhysRevLett.118.045701 28186798

[21] Wang, C. et al. Electromagnetically induced transparency at a chiral exceptional point. *Nat. Phys.* **16**, 334–340 (2020).

[22] Mao, W., Fu, Z., Li, Y., Li, F. & Yang, L. Exceptional-point-enhanced phase sensing. *Sci. Adv.* **10**, eadl5037 (2024). 10.1126/sciadv.adl5037 PMC10997194 38579005

[23] Zhen, B. et al. Spawning rings of exceptional points out of Dirac cones. *Nature* **525**, 354–358 (2015). 10.1038/nature14889 26352476

[24] Song, Q., Odeh, M., Zúñiga-Pérez, J., Kanté, B. & Genevet, P. Plasmonic topological metasurface by encircling an exceptional point. *Science* **373**, 1133–1137 (2021). 10.1126/science.abj3179 34516834

[25] Wang, C., Sweeney, W. R., Douglas Stone, A. & Yang, L. Coherent perfect absorption at an exceptional point. *Science* **373**, 1261–1265 (2021). 10.1126/science.abj1028 34516794

[26] Krasnok, A. et al. Anomalies in light scattering. *Adv. Opt. Photonics* **11**, 892 (2019).

[27] He, T. et al. Scattering exceptional point in the visible. *Light Sci. Appl.* **12**, 229 (2023). 10.1038/s41377-023-01282-4 PMC10504253 37714831

[28] Doppler, J. et al. Dynamically encircling an exceptional point for asymmetric mode switching. *Nature* **537**, 76–79 (2016). 10.1038/nature18605 27454554

[29] Zhang, X.-L., Jiang, T. & Chan, C. T. Dynamically encircling an exceptional point in anti-parity-time symmetric systems: asymmetric mode switching for symmetry-broken modes. *Light Sci. Appl.* **8**, 88 (2019). 10.1038/s41377-019-0200-8 PMC6804564 31645932

[30] Said Ergoktas, M. et al. Topological engineering of terahertz light using electrically tunable exceptional point singularities. *Science* **376**, 184–188 (2022). 10.1126/science.abn6528 PMC7612901 35389774

[31] Engheta, N. Four-dimensional optics using time-varying metamaterials. *Science* **379**, 1190–1191 (2023). 10.1126/science.adf1094 36952405

[32] Moussa, H. et al. Observation of temporal reflection and broadband frequency translation at photonic time interfaces. *Nat. Phys.* **19**, 863–868 (2023).

[33] Galiffi, E. et al. Optical coherent perfect absorption and amplification in a time-varying medium. arXiv:2410.16426 [physics], 2024.

[34] Qin, H., Zhang, Z., Wang, J. & Fleury, R. Topological hysteretic winding for temporal anti-lasing. *Nat. Commun.* **16**, 6189 (2025). 10.1038/s41467-025-61282-3 PMC12227549 40615388

[35] Zheludev, N. I. Time crystals for photonics and timetronics. *Nat. Photonics* **18**, 1123–1125 (2024).

[36] Goi, E., Zhang, Q., Chen, X., Luan, H. & Gu, M. Perspective on photonic memristive neuromorphic computing. *PhotoniX* **1**, 3 (2020).

[37] Cheng, Z., Ríos, C., Pernice, W. H. P., David Wright, C. & Bhaskaran, H. On-chip photonic synapse. *Sci. Adv.* **3**, e1700160 (2017). 10.1126/sciadv.1700160 PMC5617375 28959725

[38] Chen, M. et al. Selective and quasi-continuous switching of ferroelectric Chern insulator devices for neuromorphic computing. *Nat. Nanotechnol.* **19**, 962–969 (2024). 10.1038/s41565-024-01698-y 38965346

[39] Kim, D., Baucour, A., Choi, Y.-S., Shin, J. & Seo, M.-K. Spontaneous generation and active manipulation of real-space optical vortices. *Nature* **611**, 48–54 (2022). 10.1038/s41586-022-05229-4 36224392

[40] Wang, K., Dutt, A., Wojcik, C. C. & Fan, S. Topological complex-energy braiding of non-Hermitian bands. *Nature* **598**, 59–64 (2021). 10.1038/s41586-021-03848-x 34616054

[41] Rudner, M. S., Lindner, N. H., Berg, E. & Levin, M. Anomalous Edge States and the Bulk-Edge Correspondence for Periodically Driven Two-Dimensional Systems. *Phys. Rev. X* **3**, 031005 (2013).

[42] Qin, H., Zhang, Z., Chen, Q., Zhang, Z. & Fleury, R. Anomalous-Chern Steering of Topological Nonreciprocal Guided Waves. *Adv. Mater.* **36**, 2401716 (2024). 10.1002/adma.202401716 38697614

[43] Zhang, Z., Delplace, P. & Fleury, R. Superior robustness of anomalous non-reciprocal topological edge states. *Nature* **598**, 293–297 (2021). 10.1038/s41586-021-03868-7 PMC8514337 34646003

[44] Yin, S., Galiffi, E. & Alù, A. Floquet metamaterials. *eLight* **2**, 8 (2022).

[45] Yu, L. et al. Dirac mass induced by optical gain and loss. *Nature* **632**, 63–68 (2024). 10.1038/s41586-024-07664-x 38961303

[46] Mermin, N. D. The topological theory of defects in ordered media. *Rev. Mod. Phys.* **51**, 591–648 (1979).

[47] Qin, H. et al. Disorder-assisted real-momentum topological photonic crystal. *Nature* **639**, 602–608 (2025). 10.1038/s41586-025-08632-9 40011788

[48] Yuan, L., Lin, Q., Xiao, M. & Fan, S. Synthetic dimension in photonics. *Optica* **5**, 1396–1405 (2018).

[49] Dutt, A., Minkov, M., Williamson, I. A. D. & Fan, S. Higher-order topological insulators in synthetic dimensions. *Light Sci. Appl.* **9**, 131 (2020). 10.1038/s41377-020-0334-8 PMC7371732 32704364

[50] Huang, K., Fu, H., Watanabe, K., Taniguchi, T. & Zhu, J. High-temperature quantum valley Hall effect with quantized resistance and a topological switch. *Science* **385**, 657–661 (2024). 10.1126/science.adj3742 39024378

[51] Zhao, H. et al. Sub-picosecond topological phase transition in nonlinear exciton-polariton superlattices.* Nat. Phys.,*1–7 (2025).

