# Supplementary material for: Topological memory of nonreciprocal scattering singularities
Source: Nat Commun. 2026 Sep 25;17:11105. doi: 10.1038/s41467-026-77640-8 (PMC13696698; doi:10.1038/s41467-026-77640-8)
Supplement: Supplementary file 1 [file 41467_2026_77640_MOESM2_ESM.pdf]

Supplementary Information for Topological Memory of Nonreciprocal Scattering Singularities

Haoye Qin,[1,*] Zhe Zhang,[2] Wenjing Lv,[1] Jue Li,[1] Junda Wang,[3] Zijin Yang,[4] Qinghua Song,[1] and Romain Fleury[3,†]

[1] *Tsinghua Shenzhen International Graduate School, Tsinghua University, Shenzhen 518055, China*

[2] *Department of Electrical Engineering, Ginzton Laboratory, Stanford University, Stanford, CA 94305, USA*

[3] *Laboratory of Wave Engineering, School of Electrical Engineering, EPFL, Lausanne 1015, Switzerland*

[4] *School of Physics and Optoelectronic Engineering, Beijing University of Technology, Beijing 100124, China*

* haoye.qin@sz.tsinghua.edu.cn

† romain.fleury@epfl.ch

Supplementary Note 1. Experiments on nonlinear hysteresis

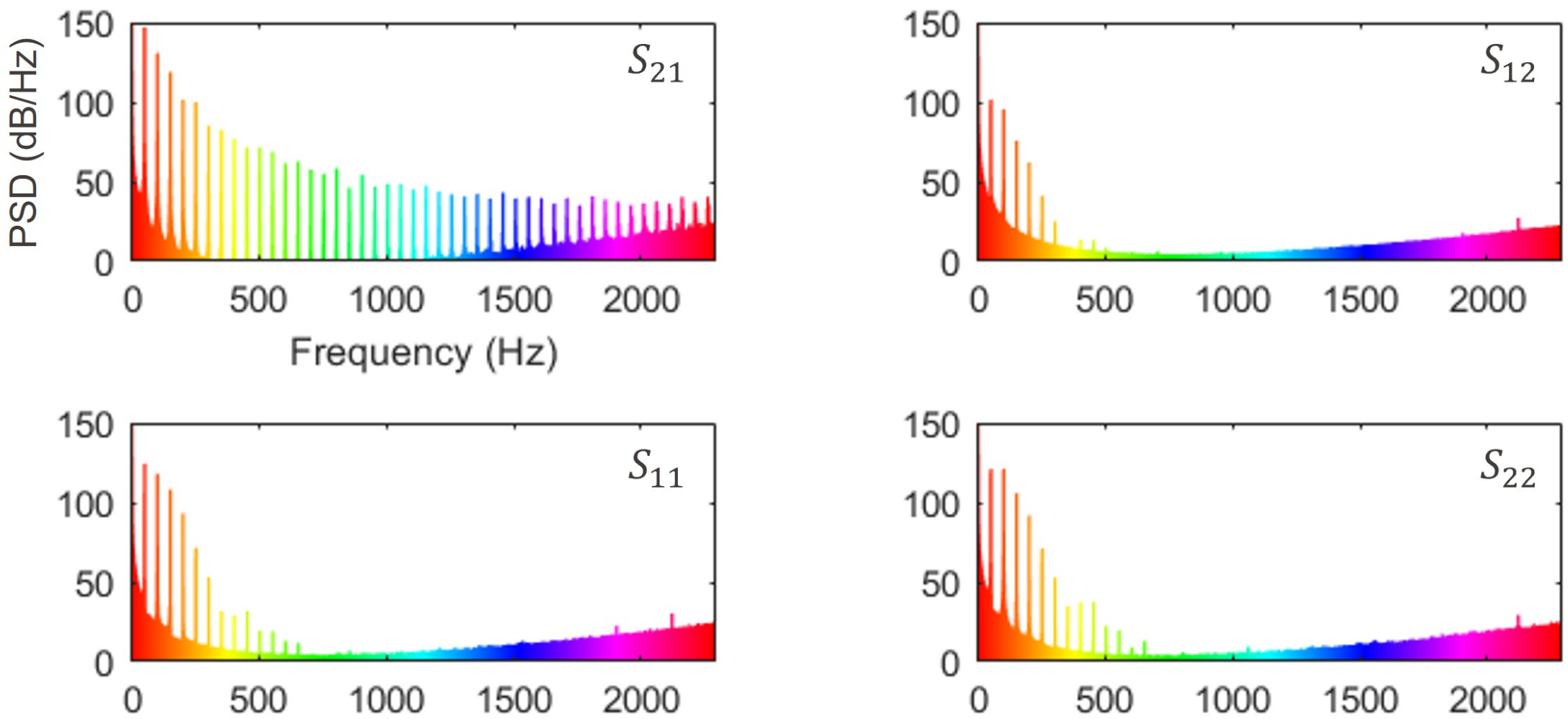

Fig. S1. **Measured hysteretic combs of scattering components.** Frequency spectrum of four scattering parameters in the nonreciprocal scattering matrix, S_{21},S_{12}, S_{11}, and S_{22}.

The frequency combs arise from nonlinear hysteresis under periodic magnetic modulation. A fixed-frequency microwave input is scattered by a time-dependent, non-sinusoidal scattering coefficient, whose Fourier components generate modulation sidebands. Near a scattering zero, amplitude collapse and rapid phase winding enhance the nonlinear temporal response, producing stronger broadband harmonics. Different spectra result from nonreciprocal forward and backward trajectories. The spectra of four components of scattering matrix in Fig. S1 reveal multiple comb orders. It's noted that S_{21} has wider combs that span over 2 kHz, indicating it is more susceptible to the temporal memory due to its vicinity near singularity. This verifies the ultrafast transition and strong harmonic generation enabled by temporal memory and the topological singularity. Although comb amplitudes may vary with loss, drift, and noise, their spacing is fixed by the modulation frequency and broadband enhancement repeatably appears near singularity crossings.

Moreover, the STFT spectrum in Fig. S2 confirms the ultrafast and strongly nonlinear phenomenon at singularity, reflecting in both phase and amplitude of S_{21}. Due to nonreciprocity, S_{12} does not encounter a singularity and therefore exhibits no higher-order transient combs. This also holds true for EP. We define the value $\delta_{EP} = S_{21} - S_{21EP}$ that

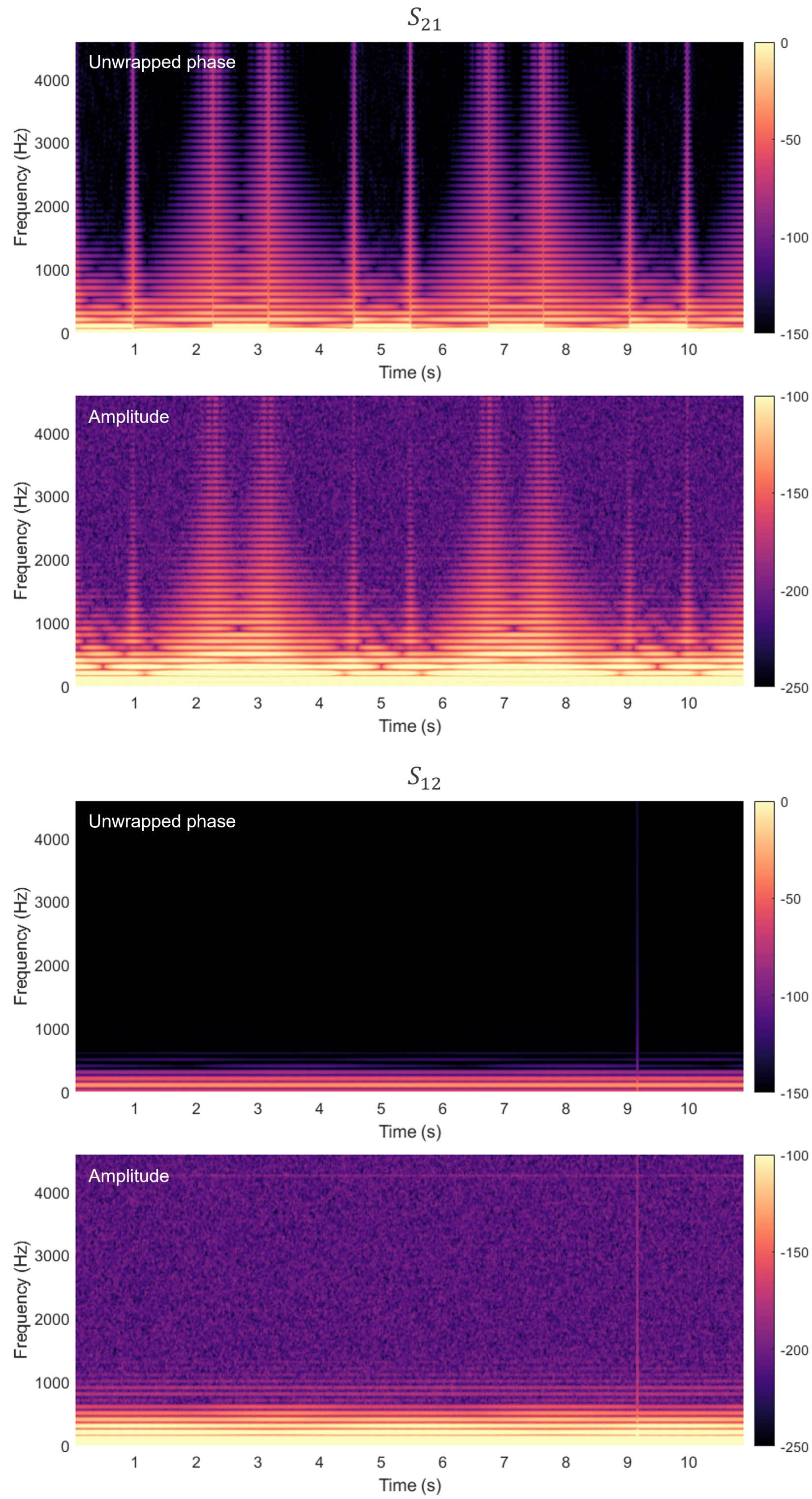

Fig. S2. **Measured short-time Fourier transformation (STFT) of S_{21} and S_{12}.** The frequency domain shows similar features between unwrapped phase and amplitude. The color scale is in dB.

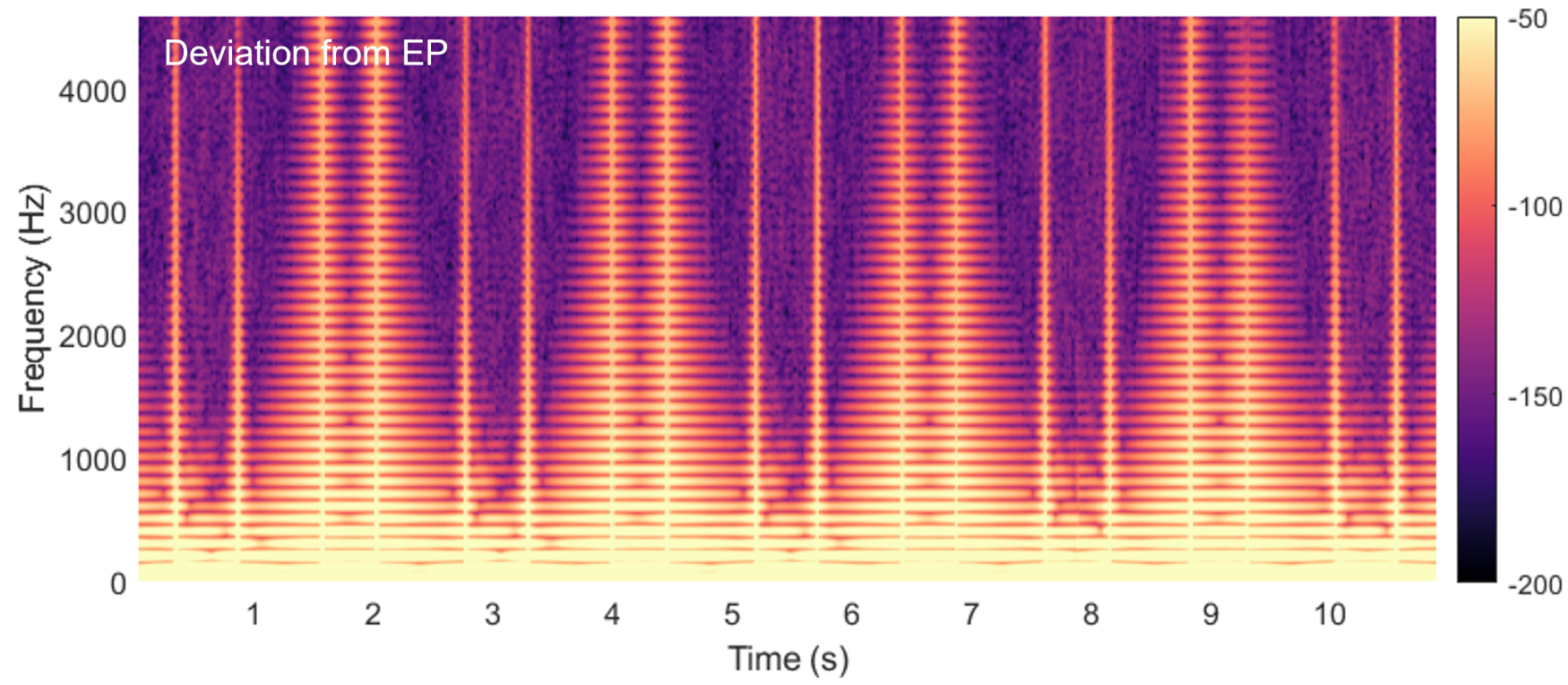

Fig. S3. **Measured short-time Fourier transformation (STFT) of deviation from EP δ_{EP}.** EP is achieved with $\delta_{EP} = 0$.

characterizes the deviation from an exact EP, where S_{21EP} is the required value of S_{21} for approaching coalesced eigenvalues. The STFT spectrum has similar features at the time of $\delta_{EP} = 0$, confirming fast EP phenomenon (Fig. S3). Generally, a scattering zero denotes a point in frequency or parameter space where a complex scattering amplitude vanishes, which is usually realized with absorption. At this point the phase is undefined, so the zero acts as a topological defect: a closed trajectory encircling it accumulates a quantized phase winding. This channel-resolved zero is closely related to coherent perfect absorption, where an eigenvalue of the full scattering matrix vanishes ($\det(S) = 0$) and a properly phased coherent input is completely absorbed.

Supplementary Note 2. Eigenstate of nonreciprocal EP

Consider a general nonreciprocal scattering matrix S where a, b, c, d are complex values

$$S = \begin{bmatrix} a & b \\ c & d \end{bmatrix}. \tag{1}$$

The eigenvalues are $\lambda_{1,2} = \dfrac{a + d \pm \sqrt{a^2 - 2ad + 4bc + d^2}}{2}$ and EP requires $(a-d)^2 + 4bc = 0$. The corresponding eigenstate at nonreciprocal EP is $v_{EP} = [\dfrac{a-d}{2c}\ 1]^T$. For reciprocal system, $b = c$ results in fixed $v_{EP} = [\pm i\ 1]^T$. For infinite nonreciprocity $c \to 0$, the eigenstate will move towards $v_{EP} = [1\ 0]^T$ (or $v_{EP} = [0\ 1]^T$ for $b \to 0$).

These EP eigenstates, given in complex column vectors, can be mapped onto Poincaré

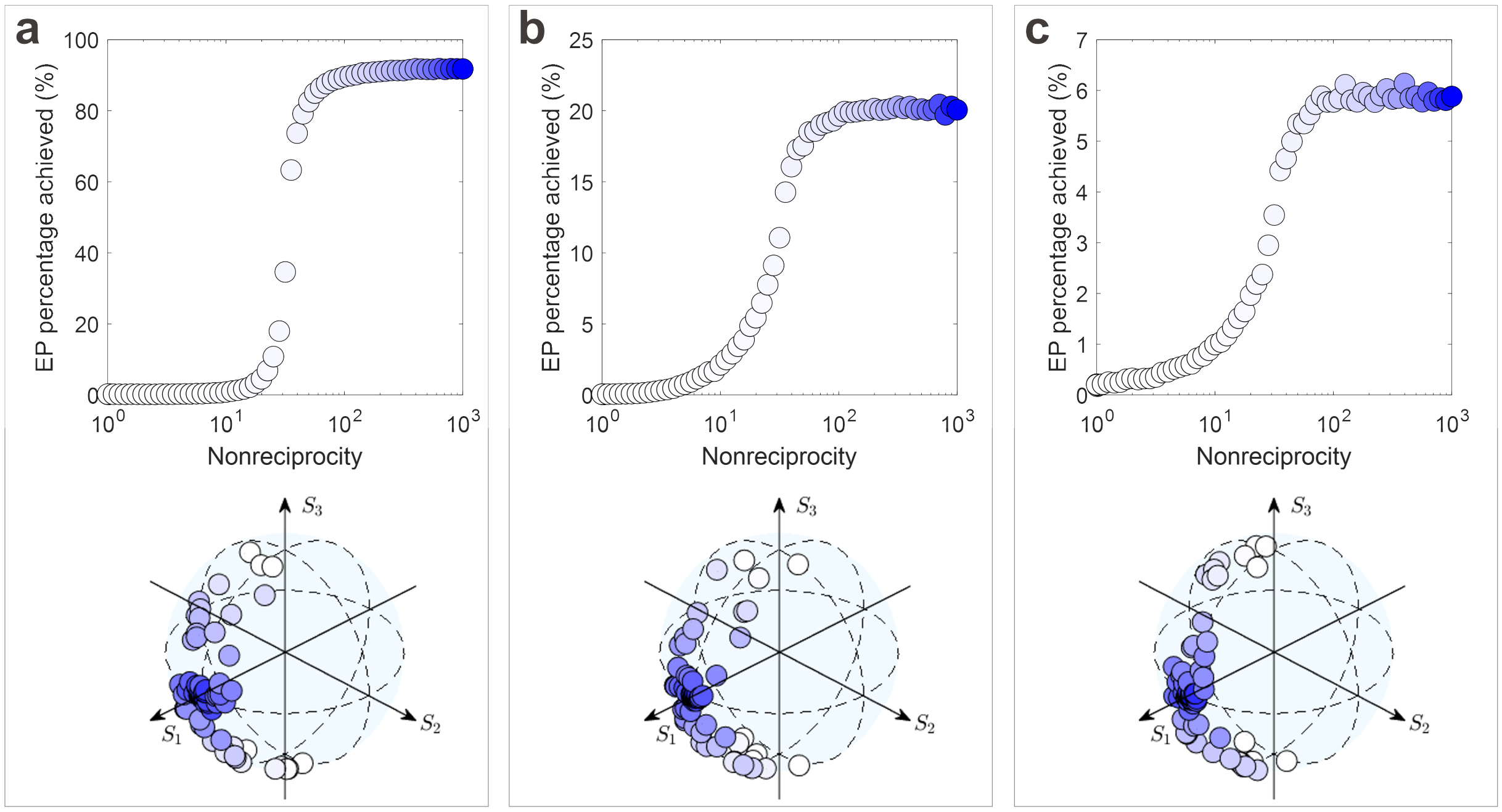

Fig. S4. **Nonreciprocity enabled easier EPs** The random scattering matrices under evaluation have **a,** smaller, **b,** medium, and **c,** larger reflection a, d. The corresponding coalesced eigenstates at EP are plotted on Poincaré sphere where reciprocal EP must locate on two poles ($[1\ \pm j]^T$) and nonreciprocal EPs span over the whole sphere. For nearly infinite nonreciprocity, EP eigenstates occur near $[1\ 0]^T$ or $[0\ 1]^T$ on the equator. The statistical results are extracted from 10^5 random scattering matrices for each value of nonreciprocity by identifying the fraction that satisfies the EP condition within the specific numerical tolerance.

sphere as polarization state. For reciprocal EPs, the eigenstates are anchored to either one of the two poles. For nonreciprocal EPs, the eigenstates can span over the full sphere. EPs with nearly infinite nonreciprocity lie close to X or Y polarization on the equator [1].

As detailed in Fig. 3a in the main text, nonreciprocity will assist in EP degeneracy, making EPs easier to realize. In Fig. S4 we evaluate the effect of different levels of reflection on this trend. We denote the nonreciprocity sweep as η, and the random-matrix ensembles used in Fig. 3a and Figs. S4a–S4c are denoted by S_1–S_4, respectively,

$$S_1 = \begin{bmatrix} a & b\eta \\ b & d \end{bmatrix}, \tag{2}$$

$$S_2 = \begin{bmatrix} a\eta & b\eta \\ b & d\eta \end{bmatrix}, \tag{3}$$

$$S_3 = \begin{bmatrix} 0.5a\eta & b\eta \\ b & 0.5d\eta \end{bmatrix}, \tag{4}$$

$$S_4 = \begin{bmatrix} 0.1a\eta & b\eta \\ b & 0.1d\eta \end{bmatrix}. \tag{5}$$

We only consider η as a real-valued nonreciprocity while complex value is also possible. In general, larger nonreciprocity will make EP occur easier. The backscattering zero facilitates the EP by producing the infinite-nonreciprocity limit; the EP is confirmed from the full measured scattering matrix through simultaneous eigenvalue coalescence and a braiding transition.

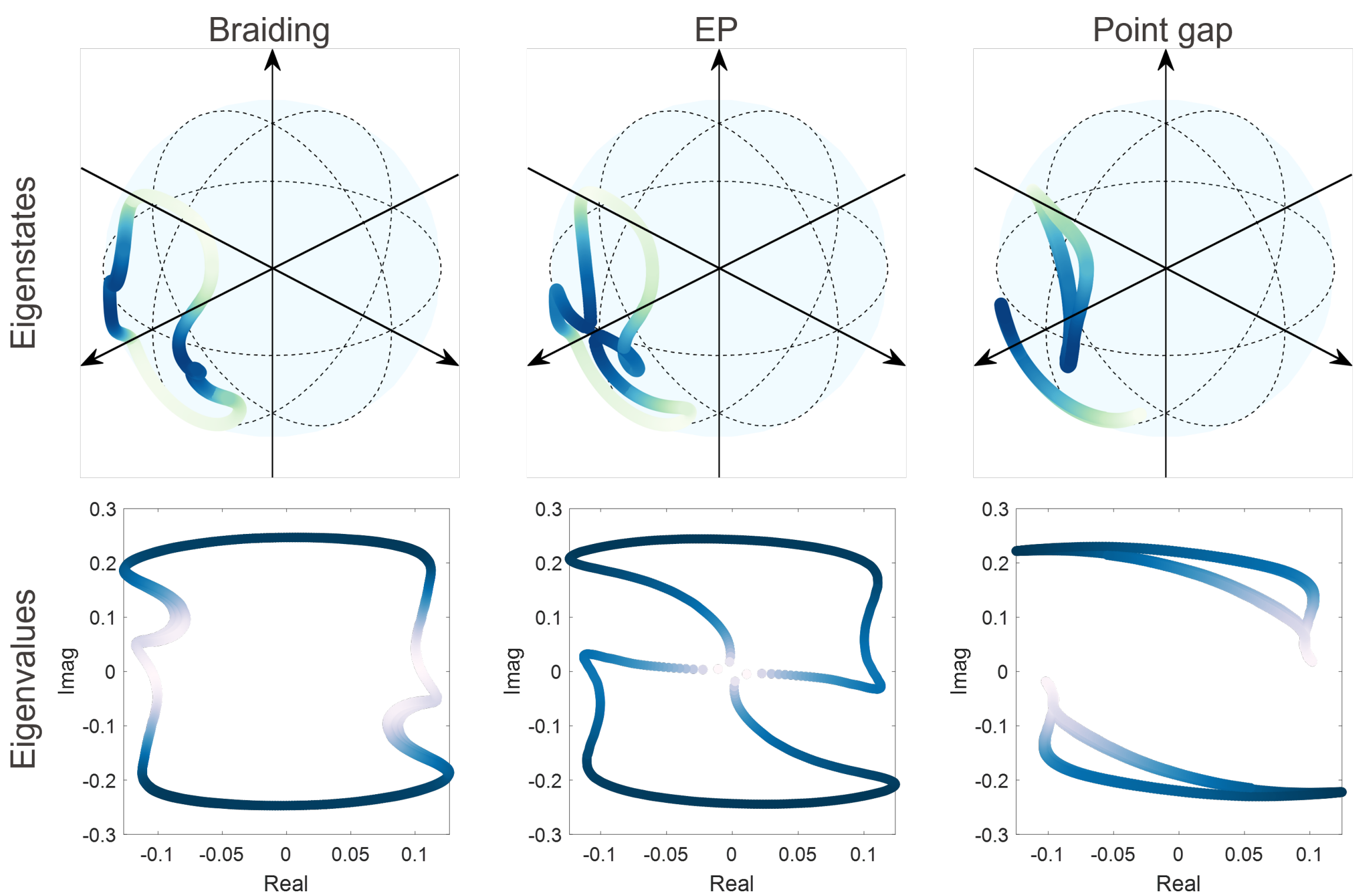

Fig. S5. **Evolution of eigenstates on Poincaré sphere and eigenvalues projected onto complex plane.** At EP, the loops of eigenvalues coalesce at one point and the EP degeneracy is near $[1\ 0]^T$.

Three typical cases of eigenstates and projected eigenvalues for non-Hermitian braiding, EP transition, and point gap are shown in Fig. S5 (in correspondence with Figs. 3d-f in

the main text.). The evolution of eigenstates in time domain also carry hysteretic memory effects (see Fig. S6), contributing to non-coalesced loops on the sphere. At EP, the loops just touch each other and the contact point is close to $[1\ 0]^T$ as predicted.

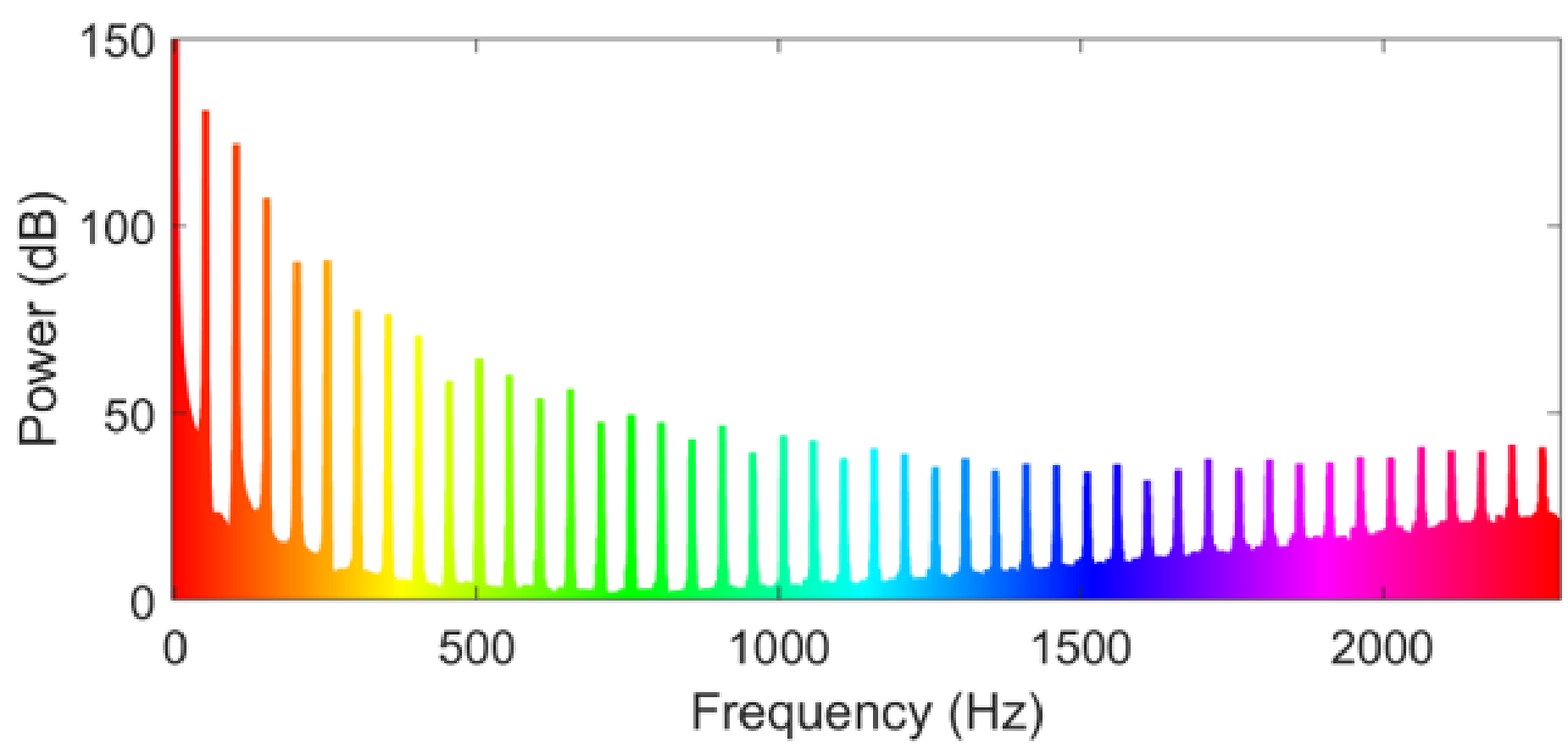

Fig. S6. **Measured hysteretic memory combs of state evolution.**

Supplementary Note 3. Braiding in time domain

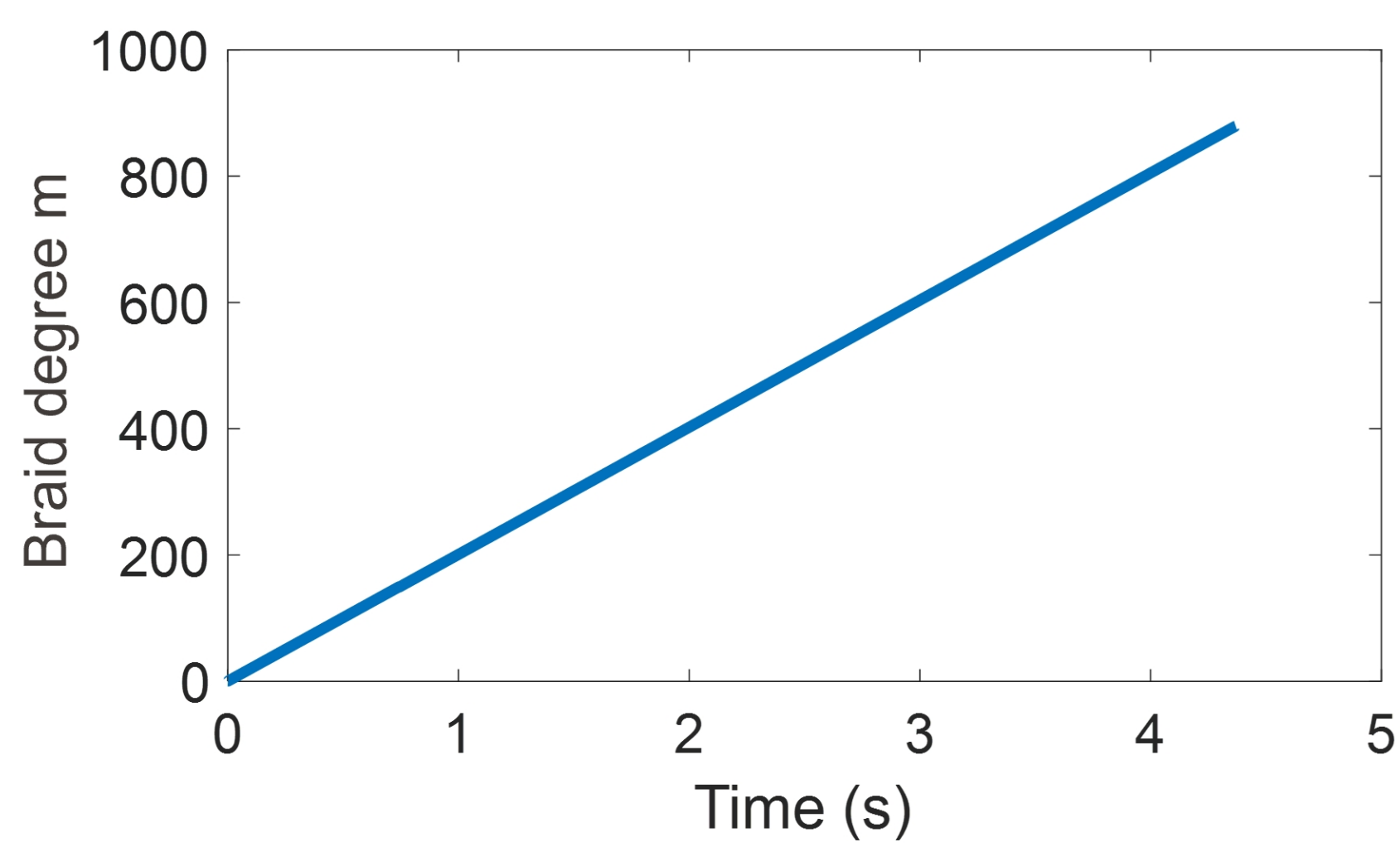

Fig. S7. **Large braid degree over time at** $f_{1,2} = 200$ **Hz.**

Extremely large braid degrees and fast braiding speed can be realized in time domain by only encircling EP using higher synchronized modulation $f_{1,2} = 200$ Hz. Within time of 4 seconds, the two non-Hermitian eigenvalues braid nearly 800 times (Fig. S7).

Supplementary Note 4. Circulator network with anomalous Floquet topology

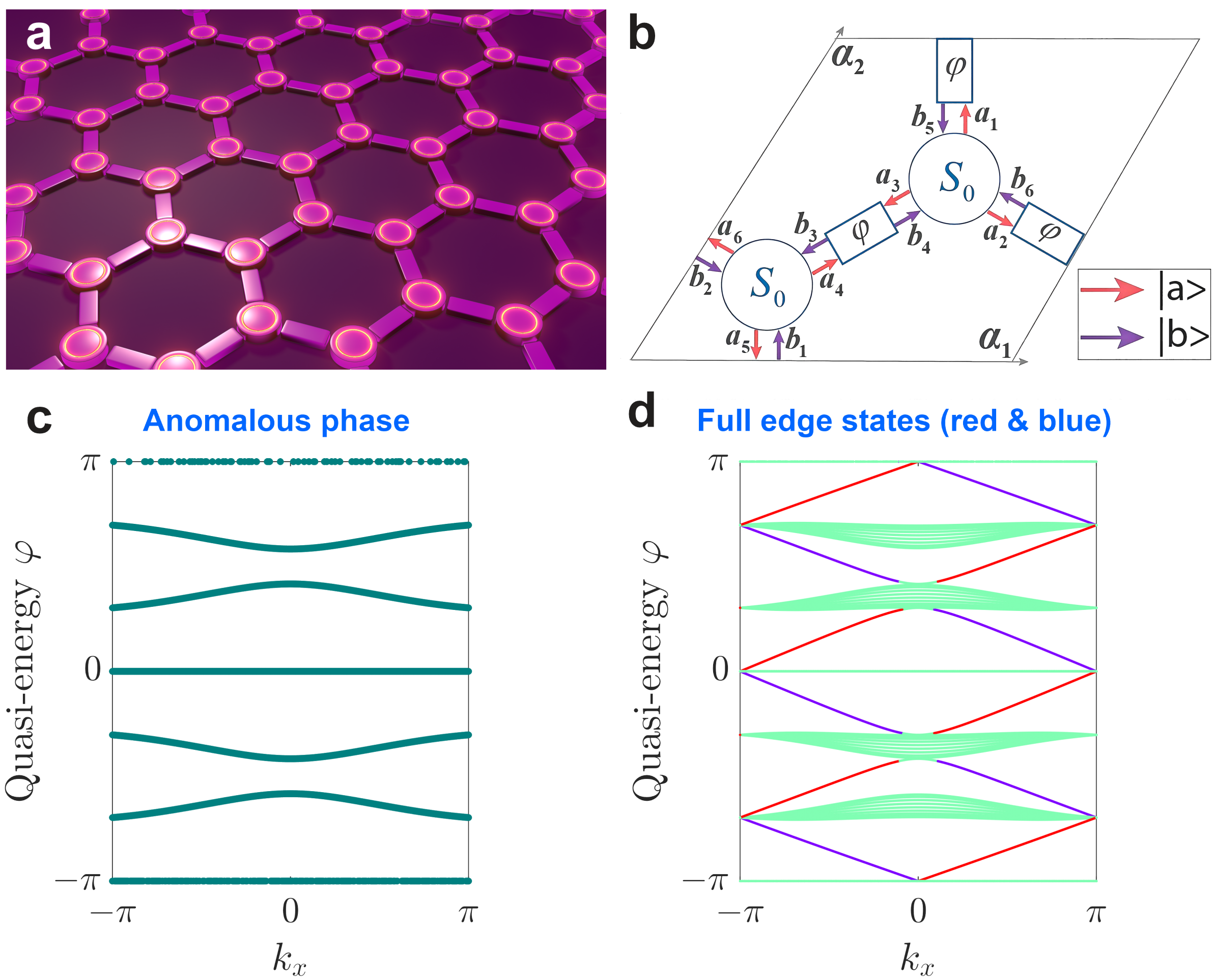

Fig. S8. **Topology of circulator network. a,** Honeycomb circulator topological insulator. **b,** Schematic of unit cell in representation of scattering matrix formalism. S_0 denotes the scattering matrix of circulator, and φ is the phase delay of transmission line. $\alpha_{1,2}$ is the periodicity. **c,** Band structure of anomalous phase for **b**. **d,** Ribbon band structure showing anomalous edge states that span all quasi-energy band gaps. The red and purple branches correspond to edge modes localized at the top and bottom boundaries, respectively.

4.1. Derivation of the Bloch eigenproblem for the circulator network

We obtain the phase-delay band structure of the periodic honeycomb scattering network by analyzing the two circulators contained within a single honeycomb unit cell. The action

of the associated scattering matrices on the complex wave amplitudes entering and exiting each circulator is given by (schematically illustrated in Figs. S8a and S8b):

$$\begin{bmatrix} a_3 \\ a_1 \\ a_2 \\ a_5 \\ a_6 \\ a_4 \end{bmatrix} = \begin{bmatrix} 0 & S_0 \\ S_0 & 0 \end{bmatrix} \begin{bmatrix} b_1 \\ b_2 \\ b_3 \\ b_4 \\ b_5 \\ b_6 \end{bmatrix} \tag{6}$$

where these vectors collect the six complex wave amplitudes incident on the two circulators within the unit cell (denoted a_1 to a_6) as well as the six outgoing amplitudes (denoted b_1 to b_6). We then left-multiply Eq. (6) by a unitary permutation matrix P_0, which successively exchanges the fifth and sixth rows, the fourth and fifth rows, and the first and second rows, yielding:

$$|a\rangle = S_{\text{unit cell}}|b\rangle \qquad \text{with} \quad S_{\text{unit cell}} \equiv P_0 \begin{bmatrix} & S_0 \\ S_0 & \end{bmatrix}, \tag{7}$$

where $|a\rangle$ and $|b\rangle$ are functions of the position $\boldsymbol{r}$ of the unit cell, with $\boldsymbol{r} = n_1\boldsymbol{\alpha_1} + n_2\boldsymbol{\alpha_2}$, where $\boldsymbol{\alpha_1}$ and $\boldsymbol{\alpha_2}$ are Bravais lattice vectors, and $\boldsymbol{n} = n_1, n_2$ are integers.

With the phase delay lines between circulators, we obtain:

$$b_1(\boldsymbol{r} + \boldsymbol{\alpha_2}) = e^{i\varphi} a_1(\boldsymbol{r}) \tag{8}$$

$$b_2(\boldsymbol{r} + \boldsymbol{\alpha_1}) = e^{i\varphi} a_2(\boldsymbol{r}) \tag{9}$$

$$b_3(\boldsymbol{r}) = e^{i\varphi} a_3(\boldsymbol{r}) \tag{10}$$

$$b_4(\boldsymbol{r}) = e^{i\varphi} a_4(\boldsymbol{r}) \tag{11}$$

$$b_5(\boldsymbol{r}) = e^{i\varphi} a_5(\boldsymbol{r} + \boldsymbol{\alpha_2}) \tag{12}$$

$$b_6(\boldsymbol{r}) = e^{i\varphi} a_6(\boldsymbol{r} + \boldsymbol{\alpha_1}). \tag{13}$$

By looking for Bloch eigenstates, we obtain

$$|a(\boldsymbol{k})\rangle = e^{-i\varphi} \Lambda(\boldsymbol{k}) |b(\boldsymbol{k})\rangle, \tag{14}$$

where $\Lambda(\boldsymbol{k}) = \text{diag}\big(e^{i\boldsymbol{k}\cdot\boldsymbol{\alpha_2}}, e^{i\boldsymbol{k}\cdot\boldsymbol{\alpha_1}}, 1, 1, e^{-i\boldsymbol{k}\cdot\boldsymbol{\alpha_2}}, e^{-i\boldsymbol{k}\cdot\boldsymbol{\alpha_1}}\big)$ is a unitary matrix. Combining Eqs. (7) and (14), we obtain the eigen-equation in momentum space formed by $S(\boldsymbol{k})$ as

$$\boxed{S(\boldsymbol{k})|b(\boldsymbol{k})\rangle = e^{-i\varphi(\boldsymbol{k})}|b(\boldsymbol{k})\rangle.} \tag{15}$$

This equation represents the Bloch eigenproblem of a scattering network built from three-port circulators, from which the band structure and the associated topological properties can be subsequently analyzed. We can see that $S(\boldsymbol{k}) = \Lambda^{-1}(\boldsymbol{k})S_{\text{unit cell}}$ is unitary, and $\varphi(\boldsymbol{k})$ is a real-valued phase defined modulo 2π. The values of $\varphi(\boldsymbol{k})$ as a function of $\boldsymbol{k}$ define the phase band structure, which is similar to that of Floquet lattices where the quasi-energy plays the role of the phase-delay.

4.2. Topological nature of the circulator network

Such unitary systems are topologically characterized by a homotopy invariant defined in each gap, W_ψ, that directly counts the net number of chiral edge states in a given gap [2], which can be classified as unitary topology.

The nonreciprocal scattering network realized here can be understood as the discrete-time analogue of an oriented Kagome lattice, whose dynamics is encoded in the unitary matrix $S(\boldsymbol{k})$ [3]. Through this correspondence, the angular parameter φ naturally plays the role of a quasi-energy in a Floquet (unitary) eigenproblem associated with a periodically driven lattice. This mapping allows our system to simultaneously exploit nonreciprocal transport and the broader topological structure characteristic of unitary (Floquet) systems.

The topological properties of unitary operators, such as evolution operators or our scattering matrix, are classified by the homotopy group $\pi_3(U(N)) = Z$, whose integer elements serve as the associated topological invariants

$$W_\psi = (24\pi^2)^{-1} \int \text{tr}(V_\psi^{-1}\text{d}V_\psi)^3 \quad \in \mathbb{Z}, \tag{16}$$

The cubic power is interpreted within the formalism of differential forms, and the integration is carried out over a three-torus spanned by momentum and the temporal coordinate t (over one period T). Although time does not explicitly appear in scattering networks, the cyclic structure allows one to introduce a continuous interpolation that effectively plays the role of time, thereby enabling a direct correspondence with a Floquet, i.e., T-periodic, evolution operator $U(t, \boldsymbol{k})$. The operator V_ψ is then defined as the corresponding periodized evolution operator. For Floquet systems, it takes the form

$$V_\psi(t, \boldsymbol{k}) = U(t, \boldsymbol{k})e^{itH_{\text{eff}}(\boldsymbol{k})}, \tag{17}$$

with

$$H_{\text{eff}}(\boldsymbol{k}) = \frac{i}{T} \ln_{-\psi} U(t = T, \boldsymbol{k}), \tag{18}$$

where $-\psi$ denotes the branch-cut of the logarithm. The procedure to define such an operator V_ψ and thus the invariant W_ψ for discrete-time evolutions, (i.e. when the dynamics is given by a succession of scattering events and where time therefore does not appear explicitly), like in our model, was developed in a previous detailed study [3]. Importantly, the branch-cut ψ must be chosen in a spectral gap of $U(T, \boldsymbol{k})$, or $S(\boldsymbol{k})$ in our case. For this reason, W_ψ is said to be a gap invariant, and indeed directly gives the number of chiral edge states in a given quasi-energy gap φ. In contrast, Chern numbers are band invariants. They are inferred from $H_{\text{eff}}(\boldsymbol{k}) = \frac{i}{T} \ln_{-\psi} U(t = T, \boldsymbol{k})$ and thus cannot capture the full unitary evolution.

4.3. Existence of anomalous topology

There are six bands in the quasi-energy spectrum. We calculate the gap invariants W_ψ for the band structures of Figs. S8c and S8d as 1,1,1,1,1,1 with anomalous edge state. The corresponding Chern numbers are 0,0,0,0,0,0.

Together with the non-zero gap invariants obtained for each quasi-energy gap, these results confirm the presence of a Floquet anomalous phase in which all band Chern numbers vanish while chiral edge states persist in every gap. Because these anomalous edge modes occupy all available gaps, they are expected to exhibit exceptional robustness against disorder in supporting nonreciprocal wave transport, as demonstrated in Fig. S9.

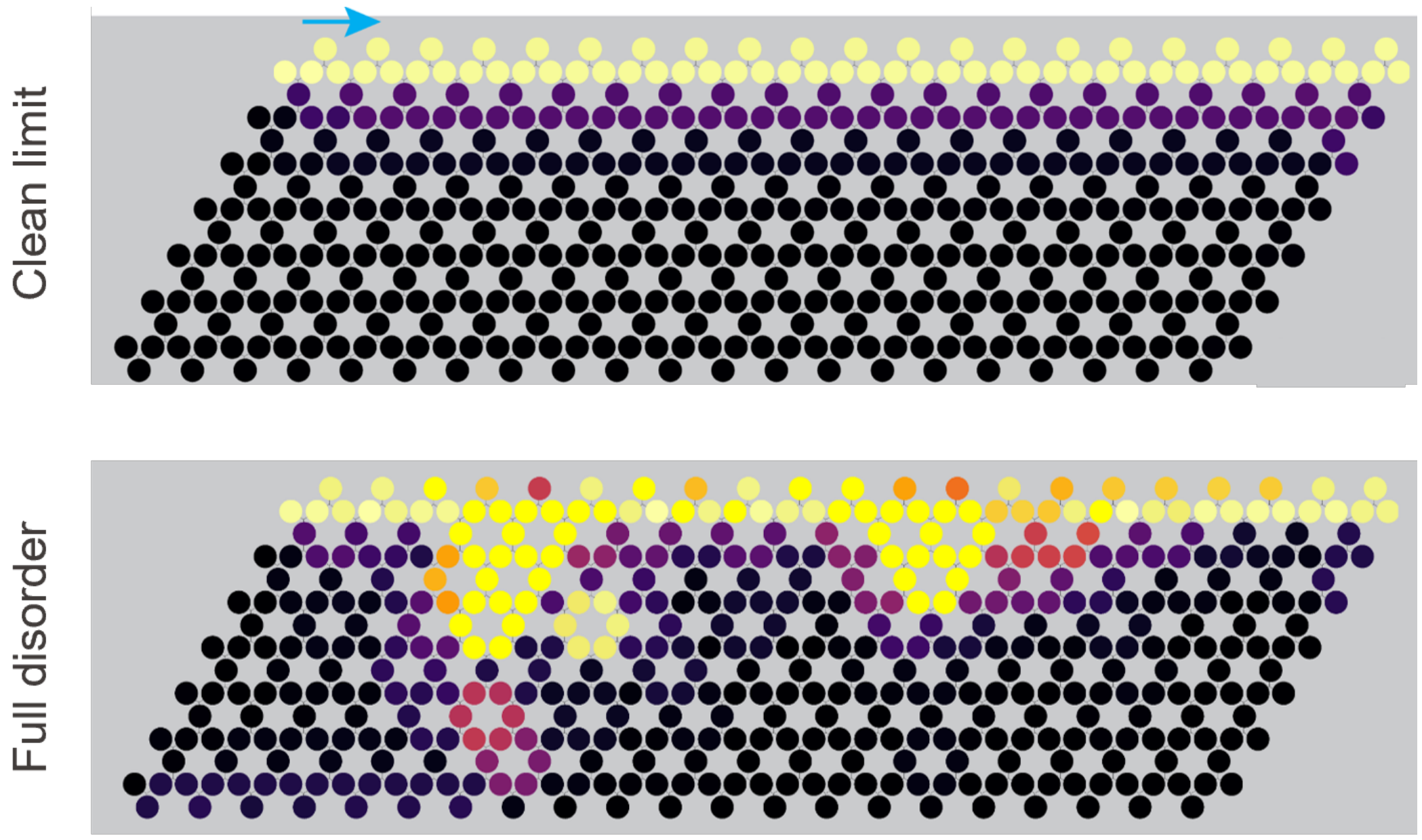

Fig. S9. **Topological anomalous edge state and its exceptional robustness.**

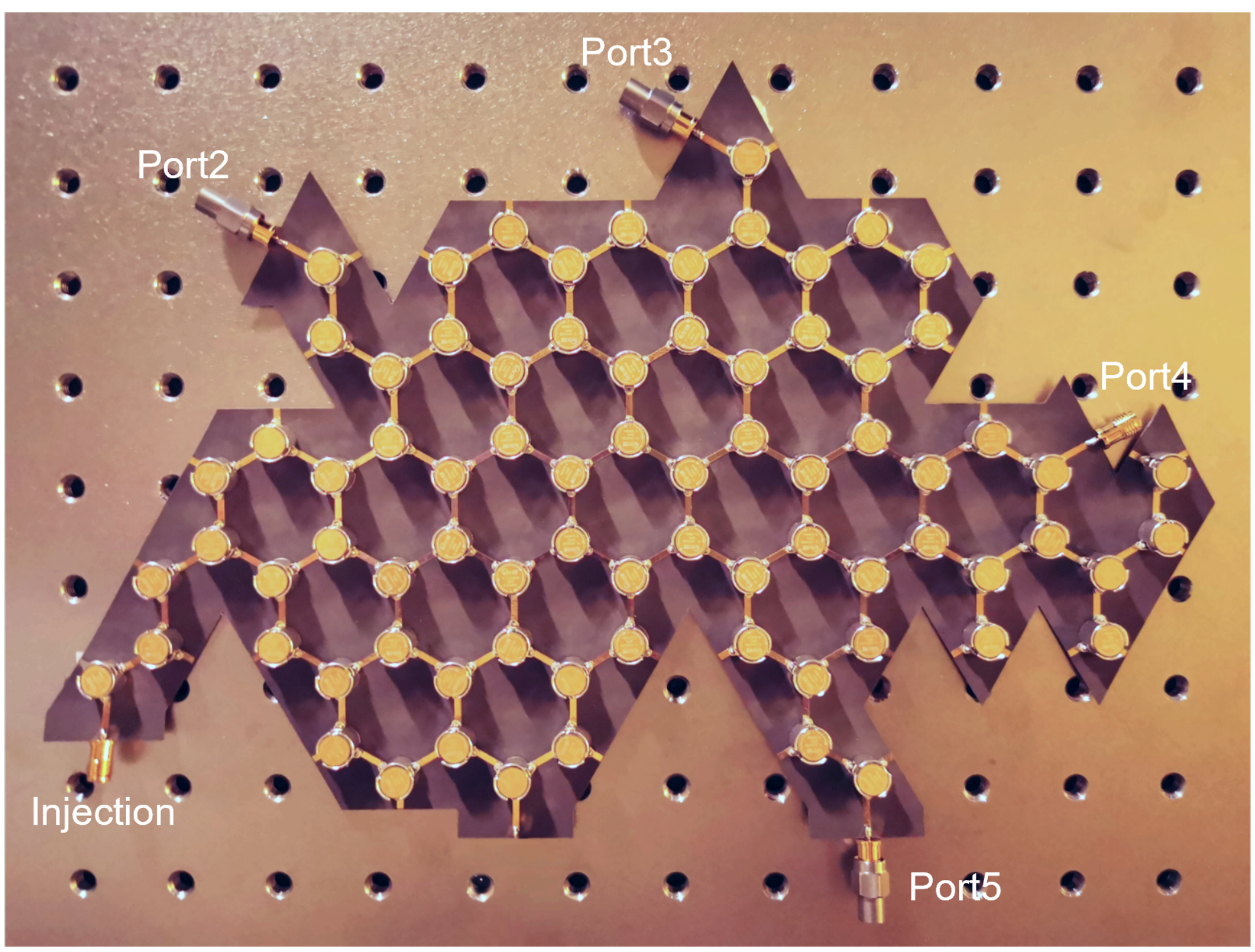

Fig. S10. **Experimental device of the topological insulator for edge-state EP transport.**

[1] M. Born and E. Wolf, Electromagnetic potentials and polarization, in *Principles of Optics: Electromagnetic Theory of Propagation, Interference and Diffraction of Light* (Cambridge University Press, Cambridge, 1999) 7th ed.

[2] M. S. Rudner, N. H. Lindner, E. Berg, and M. Levin, Anomalous Edge States and the Bulk-Edge Correspondence for Periodically Driven Two-Dimensional Systems, Physical Review X **3**, 031005 (2013).

[3] P. Delplace, M. Fruchart, and C. Tauber, Phase rotation symmetry and the topology of oriented scattering networks, Physical Review B **95**, 205413 (2017).
