# Supplementary material for: Topological memory of nonreciprocal scattering singularities
Source: Nat Commun. 2026 Sep 25;17:11105. doi: 10.1038/s41467-026-77640-8 (PMC13696698; doi:10.1038/s41467-026-77640-8)
Supplement: Supplementary file 2 [file 41467_2026_77640_MOESM3_ESM.pdf]

Description of Additional Supplementary Files

Supplementary Movie. The braiding process over time.
