# Supplementary material for: Topological memory of nonreciprocal scattering singularities
Source: Nat Commun. 2026 Sep 25;17:11105. doi: 10.1038/s41467-026-77640-8 (PMC13696698; doi:10.1038/s41467-026-77640-8)
Supplement: Supplementary file 3 [file 41467_2026_77640_MOESM4_ESM.pdf]

nature portfolio

Peer Review File

Topological Memory of Nonreciprocal Scattering Singularities

Corresponding Author: Professor Romain Fleury

This file contains all reviewer reports in order by version, followed by all author rebuttals in order by version.

Version 0:

Reviewer comments:

Reviewer #1

(Remarks to the Author)
This manuscript reports an experimental demonstration of hysteresis-induced topological memory in a nonreciprocal, non-Hermitian microwave scattering system. The temporal evolution of scattering parameters winds around nonreciprocal exceptional points (EPs). The authors show that magnetic hysteresis generates history-dependent Floquet loops in the complex scattering plane, leading to quantized winding charges, ultrafast transitions, and frequency combs. They also extended this concept of topological memory to both free-space and guided-wave teleportation of EPs within a circulator-based nonreciprocal topological insulator, demonstrating the long-distance preservation of scattering singularities assisted by nonreciprocity.

Overall, the work is technically strong, conceptually ambitious, and experimentally rigurous. I believe the manuscript is well within the scope of Nature Communications and should be of broad interest to communities working on non-Hermitian physics, topological photonics, and time-modulated systems.

Some comments the authors should address:

- The term teleportation may be viewed as potentially overstated. While the temporal EP signature is reconstructed remotely, no quantum state or information-theoretic teleportation is involved. The authors should clarify this terminology more explicitly.

- The topological memory relies on magnetic hysteresis in ferrites. It remains unclear how general the phenomenon is. Can similar effects arise in non-magnetic hysteretic systems (e.g., memristive, thermal, optomechanical)? A brief discussion of generalization beyond ferrite circulators would strengthen the manuscript.

- Are there quantitative conditions on hysteresis loop area, lag time, or loss?

- Some discussion of noise tolerance, repeatability, or parameter drift would be useful, particularly for the teleportation experiments.

Reviewer #2

(Remarks to the Author)
In the manuscript entitled, “Topological Memory of Nonreciprocal Scattering Singularities,” Qin and coworkers present an experimental configuration that enables investigation of the topological properties of a scattering zero exceptional point. By tailoring the frequencies of time-harmonic magnetic fields applied to two conjoined circulators, the system can be made to encircle the EP. The manuscript shows this via a series of short-time Fourier transforms that display frequency combs which vary in strength as a function of time. This is followed by a section titled “Topological memory of EPs” which seems to focus more on the theoretical connection between EPs and nonreciprocity rather than memory. The manuscript concludes with a section titled “Teleportation of EPs.” But this title is confusing as well as the section appears to focus more on the wireless transmission of the output of the two-circulator-system and the coupling of the output to a separate honeycomb network of circulators (neither of which is considered “teleportation”). While the experimental two-circulator setup with time-harmonic magnetic fields appears to be a useful and somewhat novel testbed for studies in topological physics, the same setup was

already put forth in a previous study by the same authors [33]. The following section, “Topological memory of EPs” is concerned with the nonreciprocal nature of the EP; however, it is unclear what connection this has with the concept of topological memory. Finally, the idea of “teleporting” an EP seems strange both due to terminology (“transmitting” would be more accurate) and because EPs are properties of systems (not signals) and generally cannot be transmitted. For these reasons, I do not recommend the manuscript for publication in Nature Communications.

A few more pointed questions related to these comments are provided in the following.

What role does nonreciprocity play in the concept of topological memory?

Fig 1(c) caption: What is the significance of 5GHz listed at the end of the caption?

Some basic model to explain the existence of frequency combs as well as the differences in spectra between the Sij would be helpful. Specifically, why do the frequency combs give evidence of backscattering zeros evolving into exceptional points?

What is the input signal for these studies? Is it a constant frequency, constant amplitude time-harmonic signal with frequency sweep?

Fig 2c: the times labeled 1-9 are not equally spaced in time; what is the significance?

Why is a statistical analysis needed to determine the number of EPs in nonreciprocal scattering matrices? Can’t this be determined using the analysis in (or similar to) Supplementary IV?

Supplementary Fig S6, How is “EP percentage achieved” determined? For 2x2 matrices, wouldn’t there be just one EP?

The presented concept of “Teleporting EPs” is unclear to me. An EP is a system property, not a property of the output signal. The diagram in Fig. 4a presumably shows the output of the circulator system being transmitted 2 meters and detected. It seems like it’s the same experimental just done but with the extra step of a wireless transmission at the output. If so, what is the significance of this demonstration?

Similar question applies to the honeycomb network. What does it mean to transmit an EP through this network? Is it simply that the output of the two-circulator system is preserved as it propagates through the network?

Minor suggestions:
Results: “…we consider a two-port scattering matrix while maintaining matched conditions for the remaining ports, thereby creating a non-unitary deficit.” Clarification, what is meant by “non-unitary deficit”. If the ports of circulator two are “matched”, are you assuming no reflection from them? Or are they open? I would think the latter since the signal is ultimately reflected back to the first circulator.

It would be useful if the Supplementary Information provided some background on the scattering zero, or CPA.

Some information on why 100Hz is chosen would be helpful.

Fig 1c: The units of PSD as dB/Hz should be clarified. How is dB defined here? Is there a reference power level, like dBm?

Supplementary Fig S3: clarify the vertical axis. Why would it be 100 dB? Unit of power (dB or dBm?). More details on how the experimental data was collected is needed.

Fig S4 and S5: what are the units of the color bars?

Supplementary Fig S10 (d) the colors look red and purple to me (not red and blue).

Pg 6: m := 1/2pi int_0^t = … Is this notation correct?

Reviewer #3

(Remarks to the Author)
In the MS titled "Topological Memory of Nonreciprocal Scattering Singularities", the authors experimentally demonstrate topological memory emerging from hysteresis-driven Floquet dynamics in a nonreciprocal microwave scattering system. Further, they demonstrate nonreciprocity-assisted tele-portation and long-distance preservation of scattering singularities. They have elaborated topological memory of exceptional points.The teleportation of nonreciprocal in memory EPs has been demonstrated. Hence, they have reported a unified experimental framework that connects singularity, nonreciprocity, topology, and Floquet engineering, enabling the realization of in-memory transient EPs and temporal topological transitions. The MS is well organized and technically sound. The findings are highly contemporary in the emerging field of non-Hermitian physics. The MS can be considered for publication once the following concerns are addressed.

1. What is the physical origin of infinite nonreciprocity?
2. The authors can comment on the possibilities of extension of the concept to non-reciprocal higher-order EPs.

3. How do the authors confirm the robustness of the combs?

Version 1:

Reviewer comments:

Reviewer #1

(Remarks to the Author)
The authors have addressed all of my comments appropriately.

Reviewer #2

(Remarks to the Author)
The authors have thoughtfully responded to my comments and revised the manuscript accordingly. I raise no further objections to publishing this manuscript.

Reviewer #3

(Remarks to the Author)
The MS can be accepted in the present form.

Open Access This Peer Review File is licensed under a Creative Commons Attribution 4.0 International License, which permits use, sharing, adaptation, distribution and reproduction in any medium or format, as long as you give appropriate credit to the original author(s) and the source, provide a link to the Creative Commons license, and indicate if changes were made.
In cases where reviewers are anonymous, credit should be given to 'Anonymous Referee' and the source.
The images or other third party material in this Peer Review File are included in the article’s Creative Commons license, unless indicated otherwise in a credit line to the material. If material is not included in the article’s Creative Commons license and your intended use is not permitted by statutory regulation or exceeds the permitted use, you will need to obtain permission directly from the copyright holder.
To view a copy of this license, visit https://creativecommons.org/licenses/by/4.0/

Dear Editors and Reviewers,

Thank you very much for your letter and for reviewers' comments concerning our manuscript entitled "*Topological Memory of Nonreciprocal Scattering Singularities*" (NCOMMS-25-98408). Those comments are all valuable and very helpful for revising and improving our paper. We have responded to these comments carefully **in purple** and have made corrections **in red** which we hope to meet with approval. The main corrections in the paper and the responds to the reviewers' comments are as follows.

Response to Reviewer #1

Comment: This manuscript reports an experimental demonstration of hysteresis-induced topological memory in a nonreciprocal, non-Hermitian microwave scattering system. The temporal evolution of scattering parameters winds around nonreciprocal exceptional points (EPs). The authors show that magnetic hysteresis generates history-dependent Floquet loops in the complex scattering plane, leading to quantized winding charges, ultrafast transitions, and frequency combs. They also extended this concept of topological memory to both free-space and guided-wave teleportation of EPs within a circulator-based nonreciprocal topological insulator, demonstrating the long-distance preservation of scattering singularities assisted by nonreciprocity.

Overall, the work is technically strong, conceptually ambitious, and experimentally rigorous. I believe the manuscript is well within the scope of Nature Communications and should be of broad interest to communities working on non-Hermitian physics, topological photonics, and time-modulated systems.

Our reply: We thank you for the positive assessment of our work and for recognizing the technical strength, conceptual scope, and experimental rigor of the manuscript.

Comment: Some comments the authors should address: The term teleportation may be viewed as potentially overstated. While the temporal EP signature is reconstructed remotely, no quantum state or information-theoretic teleportation is involved. The authors should clarify this terminology more explicitly.

Our reply: We thank you for pointing this out. We agree that the term "teleportation" could

be misleading if interpreted in the quantum-information sense. What is demonstrated is the remote reconstruction and long-distance preservation of the temporal scattering signature associated with nonreciprocal exceptional points after free-space or guided-wave propagation. We have therefore revised the terminology throughout the manuscript, replacing “teleportation” with “remote reconstruction” or “long-distance transport” where appropriate, and explicitly clarified the meaning in the Results section.

Comment: The topological memory relies on magnetic hysteresis in ferrites. It remains unclear how general the phenomenon is. Can similar effects arise in non-magnetic hysteretic systems (e.g., memristive, thermal, optomechanical)? A brief discussion of generalization beyond ferrite circulators would strengthen the manuscript.

Our reply: We thank you for raising this important point. We agree that the topological memory effect is not intrinsically limited to ferrite circulators. In our experiment, ferrites provide a convenient platform because magnetic hysteresis, loss, and nonreciprocity coexist naturally. More generally, the required ingredients are: (i) a hysteretic internal variable that makes the complex scattering response history-dependent, (ii) a non-Hermitian scattering singularity such as a zero or an EP, and (iii) a temporal modulation that drives a closed loop around or across the singularity. Therefore, related effects could also arise in memristive, thermo-optic, phase-change, optomechanical, ferroelectric, or nonlinear circuit systems. We have added a discussion to clarify this generality, while also noting that nonreciprocal EPs require a mechanism for time-reversal-symmetry breaking, which may be supplied by magnetic bias, spatiotemporal modulation, active circuits, or other nonreciprocal elements.

In the discussion section, we added:

“Although our experiment uses ferrite circulators, topological memory is not restricted to magnetic hysteresis. The key requirement is a history-dependent internal variable that drives a complex scattering parameter around a non-Hermitian singularity. Similar effects may therefore arise in memristive, thermo-optic, phase-change, optomechanical, ferroelectric, or nonlinear metamaterial systems. Ferrites are used here because hysteresis, loss, and nonreciprocity coexist naturally, while nonreciprocal EP memory in

other platforms would require an additional time-reversal-symmetry-breaking mechanism, such as spatiotemporal modulation, active circuits, or synthetic gauge fields.”

Comment: Are there quantitative conditions on hysteresis loop area, lag time, or loss?

Our reply: We thank you for this useful question. We have clarified that the topological transition is controlled mainly by whether the hysteretic trajectory in the complex scattering plane encloses or crosses a scattering singularity, rather than by a universal threshold of hysteresis area, lag time, or loss. These quantities are nevertheless important experimentally: the hysteresis-induced loop area must be large enough to enclose the singularity, the lag time must be sufficient to open a closed loop rather than a nearly retraced line, and loss is required to access non-Hermitian scattering zeros or EPs. We have added a concise statement of these conditions in the Results section.

We added a brief explanation to the concept of topological memory in Page 3:

“Practically, the loop must have sufficient area and lag to avoid collapsing into a retraced line, while loss is needed to make the scattering matrix non-unitary and allow zeros or EPs.”

Comment: Some discussion of noise tolerance, repeatability, or parameter drift would be useful, particularly for the teleportation experiments.

Our reply: We thank you for this helpful suggestion. We have added a discussion of robustness in the remote-reconstruction experiments.

For the experiment results, we added in Page 10:

“Although distortions appear in the temporal trajectories, the exceptional points at the saddle centers remain clearly observable.”

“This observation also indicates a degree of tolerance to noise and parameter drift: continuous distortions of the received trajectory do not change the winding or braiding topology unless the loop crosses the singularity or the signal-to-noise ratio becomes too low to resolve the complex trajectory. Experimentally, the main sources of variation are ferrite-bias drift, modulation instability, propagation loss, and receiver noise, but the EP signatures were repeatably observed under the same modulation conditions.”

Response to Reviewer #2

Comment: In the manuscript entitled, “Topological Memory of Nonreciprocal Scattering Singularities,” Qin and coworkers present an experimental configuration that enables investigation of the topological properties of a scattering zero exceptional point. By tailoring the frequencies of time-harmonic magnetic fields applied to two conjoined circulators, the system can be made to encircle the EP. The manuscript shows this via a series of short-time Fourier transforms that display frequency combs which vary in strength as a function of time. This is followed by a section titled “Topological memory of EPs” which seems to focus more on the theoretical connection between EPs and nonreciprocity rather than memory. The manuscript concludes with a section titled “Teleportation of EPs.” But this title is confusing as well as the section appears to focus more on the wireless transmission of the output of the two-circulator-system and the coupling of the output to a separate honeycomb network of circulators (neither of which is considered “teleportation”). While the experimental two-circulator setup with time-harmonic magnetic fields appears to be a useful and somewhat novel testbed for studies in topological physics, the same setup was already put forth in a previous study by the same authors [33]. The following section, “Topological memory of EPs” is concerned with the nonreciprocal nature of the EP; however, it is unclear what connection this has with the concept of topological memory. Finally, the idea of “teleporting” an EP seems strange both due to terminology (“transmitting” would be more accurate) and because EPs are properties of systems (not signals) and generally cannot be transmitted. For these reasons, I do not recommend the manuscript for publication in Nature Communications.

Our reply: We thank you for the critical assessment. We realize that several points were not stated clearly enough in the original manuscript. First, the present work does not claim novelty only from the two-circulator hardware platform, which was indeed introduced in our previous work. The new contribution here is the use of hysteretic Floquet trajectories to probe scattering singularities: backscattering zeros, their topological winding transitions, and their connection to nonreciprocal EPs through eigenvalue braiding. We have clarified this distinction explicitly. Second, we agree that the original “Topological memory of EPs” section did not sufficiently emphasize the connection between EPs and memory. We have

revised it to state that the EP is not only a static nonreciprocal degeneracy, but the transition point of a hysteresis-driven memory loop in the scattering matrix. The memory is encoded in the history-dependent trajectory of *s(t)*, and the EP marks the transition between braiding and non-braiding temporal topology. Third, we agree that "teleportation" is potentially misleading. We have replaced it with "remote reconstruction" and "long-distance transport of EP signatures." We now explicitly state that an EP itself is a property of an effective scattering system and is not literally transmitted as a signal.

Overall, we have revised the manuscript to clarify the novelty, strengthen the connection between memory and EP transitions, and replace "teleportation" with "remote reconstruction" or "transport of EP signatures." These changes make clear that the central result is the memory-driven topology of nonreciprocal scattering singularities, not the hardware platform alone.

Comment: A few more pointed questions related to these comments are provided in the following. What role does nonreciprocity play in the concept of topological memory?

Our reply: Nonreciprocity plays two essential roles. First, it is the natural consequence of the magnetically biased ferrite system, where time-reversal symmetry is broken and the scattering response becomes direction dependent. Second, it enables a directional scattering singularity: one transmission channel can reach a zero while the reverse channel remains finite. This produces the infinite-nonreciprocity limit, which relaxes the constraints for reaching an EP and allows the hysteretic memory loop to drive transitions between eigenvalue braiding and non-braiding regimes. We added this in Page 3:

"Nonreciprocity arises from time-reversal-symmetry breaking in magnetically biased ferrites and makes scattering direction dependent. It enables a directional zero with finite reverse transmission, creating an infinite-nonreciprocity limit that relaxes the constraints for reaching an EP and supports memory-driven braiding transitions."

Comment: Fig 1(c) caption: What is the significance of 5GHz listed at the end of the caption?

Our reply: We thank the reviewer for pointing out this ambiguity. The 5 GHz value denotes

the microwave carrier frequency at which the scattering signal is measured, whereas the comb spacing is determined by the low-frequency magnetic modulation. We intend to explicitly state that the measured signal is a microwave scattering signal.

Comment: Some basic model to explain the existence of frequency combs as well as the differences in spectra between the Sij would be helpful. Specifically, why do the frequency combs give evidence of backscattering zeros evolving into exceptional points?

Our reply: We thank the reviewer for this helpful suggestion. The frequency combs originate from the nonlinear hysteretic response of the ferrite under periodic magnetic modulation. A fixed-frequency microwave input is multiplied by the time-dependent scattering coefficient $S_{ij}(t)$; because hysteresis makes $S_{ij}(t)$ nonlinear and non-sinusoidal, its Fourier components generate comb sidebands. Near a scattering zero, the amplitude collapse and rapid phase winding further enhance this nonlinearity, producing stronger broadband harmonics. Different $S_{ij}(t)$ spectra arise from the directional response of the nonreciprocal system. We also clarify that combs themselves do not prove an EP; rather, the backscattering zero facilitates the EP by creating the infinite-nonreciprocity limit, while the EP is confirmed from eigenvalue coalescence and braiding transition of the full scattering matrix.

We have clarified this in the Supplementary Materials:

"The frequency combs arise from nonlinear hysteresis under periodic magnetic modulation. A fixed-frequency microwave input is scattered by a time-dependent, non-sinusoidal scattering coefficient, whose Fourier components generate modulation sidebands. Near a scattering zero, amplitude collapse and rapid phase winding enhance the nonlinear temporal response, producing stronger broadband harmonics. Different spectra result from nonreciprocal forward and backward trajectories."

"The backscattering zero facilitates the EP by producing the infinite-nonreciprocity limit; the EP is confirmed from the full measured scattering matrix through simultaneous eigenvalue coalescence and a braiding transition."

Comment: What is the input signal for these studies? Is it a constant frequency, constant

amplitude time-harmonic signal with frequency sweep?

Our reply: We thank you for asking us to clarify the excitation condition. In the time-domain measurements used to reconstruct the memory trajectories and frequency combs, the input is a continuous-wave microwave signal with fixed carrier frequency and constant amplitude, generated by the vector network analyzer. The carrier frequency is selected near the operating frequency of the circulator system, typically 5 GHz. The time dependence arises from the low-frequency magnetic modulation applied to the circulators, not from a frequency sweep of the microwave input.

We mentioned this explicitly in the methods:

"Two ports are connected to a vector network analyzer (VNA, Rohde & Schwarz), with all other ports terminated by matched loads. For time-domain measurements, the VNA provides a constant-amplitude continuous-wave microwave input at a fixed carrier frequency, typically near 5 GHz, while temporal variation is produced by low-frequency magnetic modulation over the circulator."

Comment: Fig 2c: the times labeled 1-9 are not equally spaced in time; what is the significance?

Our reply: We thank the reviewer for pointing this out. The labels 1-9 denote topological transition points where the hysteretic memory loop crosses the backscattering zero $S_{21} = 0$, switching between trivial and topological winding regimes. They are not equally spaced because the two asynchronous modulations slowly deform the memory loop. Labels 1-4 form one beat period of the evolving loop, while later labels correspond to the following beat periods.

We noted this in the caption:

"The numbers 1-9 mark topological transition points where the hysteretic memory loop crosses the backscattering zero, switching between trivial and topological winding regimes."

Comment: Why is a statistical analysis needed to determine the number of EPs in nonreciprocal scattering matrices? Can't this be determined using the analysis in (or similar to) Supplementary IV?

Our reply: We thank the reviewer for raising this point. EPs are determined analytically for any given scattering matrix, as derived in Supplementary Section IV and used in our analysis. The statistical analysis in Fig. 3a is not used to identify EPs, but to visualize that *nonreciprocity reduces the parameter fine tuning required to reach an EP*. In reciprocal two-port scattering, the EP condition is more restrictive, and the EP eigenstates are pinned to circular states. Breaking reciprocity lifts these restrictions, allowing EP eigenstates to span the Poincaré sphere and making EPs easier to access, especially near the infinite-nonreciprocity limit.

We have added in Page 6:

"In Fig. 3a, we run a statistical study involving generating random complex scattering matrices with different values of nonreciprocity and determining the number of EPs achieved in these random matrices. The EP condition for a given matrix is obtained analytically in Supplementary Section IV and used in our analysis. We find that higher nonreciprocity contributes to matrix degeneracy and an easier realization of EP, in contrast with reciprocal systems (see Supplementary Section IV for detailed model and effects of reflection on the trend)."

Comment: Supplementary Fig S6, How is "EP percentage achieved" determined? For 2x2 matrices, wouldn't there be just one EP?

Our reply: "EP percentage achieved" is extracted from 10^5 random scattering matrices for each prescribed nonreciprocity level. It is the fraction of matrices that satisfy the EP condition within the numerical tolerance, not the number of EPs in one matrix. We have clarified this in the revised caption/text.

"The statistic results are extracted from 10^5 random scattering matrices for each value of nonreciprocity."

Comment: The presented concept of "Teleporting EPs" is unclear to me. An EP is a system property, not a property of the output signal. The diagram in Fig. 4a presumably shows the output of the circulator system being transmitted 2 meters and detected. It seems like it's the same experiment just done but with the extra step of a wireless transmission at the

output. If so, what is the significance of this demonstration?

Our reply: We thank the reviewer for raising this point. We agree that an EP is a property of a scattering matrix, not of an output signal, and we have revised the terminology accordingly. Fig. 4 does not claim literal transmission of an EP; instead, it shows that the time-dependent complex scattering trajectory encoding the EP topology can be carried through free-space or guided-wave channels and reconstructed remotely. This is nontrivial because the signature depends on amplitude, phase, eigenvalue coalescence, and braiding, which may be degraded by loss, distortion, or channel imbalance. Physically, it demonstrates that non-Hermitian topological memory is not restricted to a local device, and suggests possible uses in remote readout, distributed sensing, and information encoding based on robust temporal scattering trajectories.

We have replaced "teleportation" with "remote reconstruction" or "transport of EP" and clarified this point in the manuscript in Page 8:

"Finally, we demonstrate remote reconstruction and transport of nonreciprocal in-memory EP signatures. An EP is a property of an effective scattering matrix rather than of the propagating signal itself. Here, the time-dependent complex scattering response encoding the EP topology is transmitted through free space or a guided-wave network and reconstructed remotely. This shows that non-Hermitian topological memory can persist beyond a local device, suggesting potential applications in remote readout, distributed sensing, and information encoding based on temporal scattering trajectories."

Comment: Similar question applies to the honeycomb network. What does it mean to transmit an EP through this network? Is it simply that the output of the two-circulator system is preserved as it propagates through the network?

Our reply: We thank the reviewer for the clarification request. We agree that an EP is not a signal propagating through the honeycomb network. What is transported is the complex temporal scattering trajectory generated by the two-circulator memory system. The key significance is that this trajectory is not simply observed as an output waveform; its amplitude and phase remain sufficiently preserved to reconstruct an effective scattering matrix whose eigenvalues still exhibit EP-related coalescence and braiding at distant ports.

This is nontrivial because the network is intentionally lossy and disordered, and the ordinary transmission amplitude becomes nearly undetectable at remote ports. The recovery of the EP-type topology therefore shows that the nonreciprocal topological edge channel preserves the directional complex scattering signature, enabling long-distance readout of non-Hermitian memory topology rather than merely transmitting microwave power.

We added this significance in Page 10:

"The experiment tests whether the complex temporal scattering trajectory that defines the EP topology can survive propagation through an independent, lossy, and disordered system. Although ordinary transmission decays strongly at remote ports, the nonreciprocal edge channel preserves the directional amplitude and phase response sufficiently to recover the EP-type eigenvalue coalescence and braiding, enabling long-distance readout of non-Hermitian memory topology."

Comment: Minor suggestions: Results: "…we consider a two-port scattering matrix while maintaining matched conditions for the remaining ports, thereby creating a non-unitary deficit." Clarification, what is meant by "non-unitary deficit". If the ports of circulator two are "matched", are you assuming no reflection from them? Or are they open? I would think the latter since the signal is ultimately reflected back to the first circulator.

Our reply: We thank the reviewer for pointing out the unclear wording. The remaining circulator ports are terminated by 50-Ω matched loads, not left open. This is the standard practice for scattering-parameter measurements of multiport microwave components, because all ports not connected to the VNA should be impedance matched to avoid uncontrolled reflections. Thus, we do not rely on reflections from these ports. Signals coupled into the terminated ports are absorbed and removed from the reduced two-port description. By "non-unitary deficit," we meant that the measured two-port scattering matrix is an effective submatrix of a larger multiport lossy system; power can leave the measured two-port channels through intrinsic material loss and the matched auxiliary ports. We have revised the wording to avoid ambiguity in Page 3:

"The system is connected through transmission lines and described by an effective two-

port scattering matrix, with all auxiliary circulator ports terminated by 50-Ω matched loads as in standard multiport scattering measurements. These matched ports suppress uncontrolled reflections, making the reduced two-port matrix non-unitary together with intrinsic loss."

Comment: It would be useful if the Supplementary Information provided some background on the scattering zero, or CPA.

Our reply: We thank you for this suggestion. We have added a short discussion explaining scattering zero and CPA in the Supplementary Materials:

"Generally, a scattering zero denotes a point in frequency or parameter space where a complex scattering amplitude vanishes, e.g., $S_{21} = 0$, which is usually realized with full absorption. At this point the phase is undefined, so the zero acts as a topological defect: a closed trajectory encircling it accumulates a quantized phase winding. This channel-resolved zero is closely related to coherent perfect absorption, where an eigenvalue of the full scattering matrix vanishes (det(S) = 0) and a properly phased coherent input is completely absorbed."

Comment: Some information on why 100Hz is chosen would be helpful.

Our reply: We thank the reviewer for this helpful suggestion. The modulation frequency of 100 Hz was chosen as a practical compromise. It is slow enough for the ferrite magnetization to follow the applied field and produce a stable hysteresis loop, while fast enough to generate clearly resolved sidebands and to collect multiple memory cycles within a short measurement time. We have clarified this in the manuscript, in Page 5:

"which is chosen as a practical compromise: slow enough to ensure stable hysteresis, yet fast enough to capture multiple memory cycles within the measurement window."

Comment: Fig 1c: The units of PSD as dB/Hz should be clarified. How is dB defined here? Is there a reference power level, like dBm?

Our reply: We thank you for pointing out this ambiguity. The PSD in Fig. 1c is calculated from the measured complex scattering coefficient $S_{21}(t)$ using MATLAB periodogram with

the sampling rate specified. Since S_{21} is dimensionless, the linear PSD has units of $|S_{21}|^2/\mathrm{Hz}$. The dB scale is therefore defined as $10\log_{10}(\mathrm{PSD})$, i.e., a logarithmic scale of the scattering-coefficient PSD per Hz. It is not referenced to 1 mW and should not be interpreted as dBm.

Comment: Supplementary Fig S3: clarify the vertical axis. Why would it be 100 dB? Unit of power (dB or dBm?). More details on how the experimental data was collected is needed.

Our reply: We thank you for pointing out this ambiguity. The vertical axis in Supplementary Fig. S3 is the relative power spectral density of the measured complex scattering coefficient $S_{21}(t)$, calculated using MATLAB periodogram with the sampling rate specified. Since S_{21} is a dimensionless scattering coefficient and the spectrum is not absolutely calibrated to microwave power, the unit is relative dB/Hz rather than dBm. We have revised the axis label and caption accordingly.

Comment: Fig S4 and S5: what are the units of the color bars?

Our reply: We thank you for pointing out this missing information. The color bars in Supplementary Figs. S4 and S5 represent the power spectral density of the measured microwave scattering signal, plotted in dB. We have added the units to the corresponding captions.

Comment: Supplementary Fig S10 (d) the colors look red and purple to me (not red and blue).

Our reply: We thank you for pointing this out. We have revised the label/caption from "red and blue" to "red and purple" to match the plotted colors.

Comment: Pg 6: m := 1/2pi int_0^t = … Is this notation correct?

Our reply: We are sorry for this. We have corrected the notation.

Response to Reviewer #3

Comment: In the MS titled "Topological Memory of Nonreciprocal Scattering Singularities",

the authors experimentally demonstrate topological memory emerging from hysteresis-driven Floquet dynamics in a nonreciprocal microwave scattering system. Further, they demonstrate nonreciprocity-assisted tele-portation and long-distance preservation of scattering singularities. They have elaborated topological memory of exceptional points.The teleportation of nonreciprocal in memory EPs has been demonstrated. Hence, they have reported a unified experimental framework that connects singularity, nonreciprocity, topology, and Floquet engineering, enabling the realization of in-memory transient EPs and temporal topological transitions. The MS is well organized and technically sound. The findings are highly contemporary in the emerging field of non-Hermitian physics. The MS can be considered for publication once the following concerns are addressed.

Our reply: We thank you for the positive evaluation of our manuscript and for recognizing the technical soundness and timeliness of the work. We have addressed the concerns point by point below and revised the manuscript accordingly.

Comment: What is the physical origin of infinite nonreciprocity?

Our reply: We thank you for requesting clarification. In our system, "infinite nonreciprocity" refers to the limiting condition where one transmission coefficient vanishes while the opposite transmission channel remains finite, e.g., $S_{21} \to 0$ and $S_{12} \neq 0$. Physically, this arises from the biased ferrite circulator, where the static magnetic field lifts the degeneracy of the two counter-propagating microwave modes and produces directional circulation. Together with loss and impedance mismatch, this directional response allows destructive interference in one propagation channel, producing a backscattering zero, while the reverse channel remains transmissive. The nonreciprocity ratio therefore diverges at the zero of one transmission channel. We have clarified this in the manuscript, in Page 6:

"The backscattering zero has another implication in a nonreciprocal scattering system: it corresponds to the limit of infinite nonreciprocity, where one transmission coefficient vanishes while the opposite channel remains finite, e.g., $S_{21} \to 0$ and $S_{12} \neq 0$. Physically, the biased ferrite circulator lifts the degeneracy of counter-propagating modes and creates directional circulation; together with loss and impedance mismatch, this allows destructive

interference in one channel while maintaining finite reverse transmission."

Comment: The authors can comment on the possibilities of extension of the concept to non-reciprocal higher-order EPs.

Our reply: We thank you for this insightful suggestion. We agree that extending the present concept to nonreciprocal higher-order EPs is a promising direction. In principle, if a hysteretic nonreciprocal system contains a higher-order EP, the memory loop of the full scattering matrix could encircle or cross the corresponding higher-order degeneracy, leading to higher-order eigenvalue permutations and richer braid topology. Nonreciprocity may be especially useful because it relaxes the constraints imposed by reciprocal scattering matrices and increases the accessible space of EP eigenstates. Experimentally, such extensions could be pursued using multiport circulator networks, coupled resonator arrays, or active/spatiotemporally modulated circuits. We have added a brief comment in the Discussion:

"Another promising direction is to extend hysteresis-induced topological memory to nonreciprocal higher-order EPs. In such systems, a memory loop of the full scattering matrix could encircle or cross a higher-order degeneracy, producing higher-order eigenvalue permutations and more complex braid topology. Nonreciprocity may facilitate this extension by enlarging the accessible space of EP eigenstates, for example in multiport circulator networks, coupled resonator arrays, or active spatiotemporally modulated circuits."

Comment: How do the authors confirm the robustness of the combs?

Our reply: We thank you for this question. The robustness of the combs is confirmed by their repeatable occurrence at the modulation harmonics and by their reproducible enhancement whenever the hysteretic trajectory approaches or crosses a scattering singularity. We emphasize that the detailed comb amplitudes are not topologically protected and can vary with loss, bias drift, and noise. The robust feature is the correlation between singularity crossing/topological transition and broadband harmonic enhancement, with comb spacing fixed by the modulation frequency. We have clarified this point in the

manuscript. In Supplementary Materials, we added:

"This verifies the ultrafast transition and strong harmonic generation enabled by temporal memory and the topological singularity. Although comb amplitudes may vary with loss, drift, and noise, their spacing is fixed by the modulation frequency and broadband enhancement repeatably appears near singularity crossings."

Therefore, we have fully addressed all the concerns raised by the three reviewers.

Reviewer #1 (Remarks to the Author):

The authors have addressed all of my comments appropriately.

Reviewer #2 (Remarks to the Author):

The authors have thoughtfully responded to my comments and revised the manuscript accordingly. I raise no further objections to publishing this manuscript.

Reviewer #3 (Remarks to the Author):

The MS can be accepted in the present form.

Our reply: We are glad that all three reviewers recommend publication.
